# Analysis of Mitochondrial haemoglobin in Parkinson's disease brain

**DOI:** 10.1016/j.mito.2016.05.001

**Published:** 2016-07

**Authors:** Freya Shephard, Oliver Greville-Heygate, Susan Liddell, Richard Emes, Lisa Chakrabarti

**Affiliations:** a University of Nottingham, Faculty of Medicine, SVMS, Sutton Bonington Campus, LE12 5RD, England, UK; b Division of Animal Sciences, School of Biosciences, Sutton Bonington Campus, LE12 5RD, England, UK

**Keywords:** *Pcd*^*5j*^, Purkinje Cell Degeneration mouse strain 5j, PM, post mortem, HbA, alpha haemoglobin, HbB, beta haemoglobin, BSA, bovine serum albumin, TBS-T, tris buffered saline–tween, COXIV, cytochrome c oxidase IV, HSP90, heat shock protein 90, VDAC, voltage dependent anion channel, NDUFS3, NADH dehydrogenase ubiquinone FeS, SMAC, second mitochondrial activator of caspase, Parkinson's disease, Mitochondria, Haemoglobin, Gender, Cerebellum, Hypoxia

## Abstract

Mitochondrial dysfunction is an early feature of neurodegeneration. We have shown there are mitochondrial haemoglobin changes with age and neurodegeneration. We hypothesised that altered physiological processes are associated with recruitment and localisation of haemoglobin to these organelles.

To confirm a dynamic localisation of haemoglobin we exposed *Drosophila melanogaster* to cyclical hypoxia with recovery. With a single cycle of hypoxia and recovery we found a relative accumulation of haemoglobin in the mitochondria compared with the cytosol. An additional cycle of hypoxia and recovery led to a significant increase of mitochondrial haemoglobin (p < 0.05). We quantified ratios of human mitochondrial haemoglobin in 30 Parkinson's and matched control human post-mortem brains. Relative mitochondrial/cytosolic quantities of haemoglobin were obtained for the cortical region, substantia nigra and cerebellum. In age matched post-mortem brain mitochondrial haemoglobin ratios change, decreasing with disease duration in female cerebellum samples (n = 7). The change is less discernible in male cerebellum (n = 18). In cerebellar mitochondria, haemoglobin localisation in males with long disease duration shifts from the intermembrane space to the outer membrane of the organelle.

These new data illustrate dynamic localisation of mitochondrial haemoglobin within the cell. Mitochondrial haemoglobin should be considered in the context of gender differences characterised in Parkinson's disease. It has been postulated that cerebellar circuitry may be activated to play a protective role in individuals with Parkinson's. The changing localisation of intracellular haemoglobin in response to hypoxia presents a novel pathway to delineate the role of the cerebellum in Parkinson's disease.

## Highlights

• Intracellular Haemoglobin changes mitochondrial localisation in response to environmental cues such as hypoxia and Parkinson’s disease.
• There are molecular changes in mitochondria extracted from the cerebellum of Parkinson’s Disease brain.
• We find that mitochondrial Hb may be regulated differently according to gender.

## 1 Introduction

Parkinson's disease is most frequently a sporadic occurrence with the major risk factor being advanced age. Although a collection of symptoms define this disease, cardinal features correspond to a movement disorder including a resting tremor, rigidity and difficulties with gait and balance. Despite substantial efforts, the cause of idiopathic Parkinson's disease still remains unknown and treatment offers only symptomatic relief over a limited period of time. In recent years Parkinson's disease research has centred increasingly around the mitochondrial organelle. Mitochondrial dysfunction is a feature of Parkinson's disease with many aspects of this organelle having been implicated in disease generation and progression (Chu, 1802). Parkinson's disease is associated with mutations of the protein alpha-synuclein which is also found accumulated in Lewy bodies – the pathological hallmark of this disease (Spillantini et al., 1997). Recently it has been suggested that mitochondrial haemoglobin in neurons is reduced by an interaction with alpha-synuclein, providing further evidence that mitochondrial haemoglobin is important when thinking about Parkinson's disease (Yang et al., 2016). Altered haemoglobin levels affect the expression of genes involved in mitochondrial function, demonstrating a link between mitochondria and haemoglobin (Chuang et al., 2012). Hypoxia, due to reduced blood perfusion, is linked to ageing (Daulatzai, 2013). This is particularly relevant to brain ageing and neurodegeneration, as the brain is most sensitive to reduced oxygen levels. Hypoxia affects mitochondrial structure and function as well as intracellular haemoglobin expression (Gleixner et al., 2012). Haemoglobin (HBA2) expression changes are now also implicated in preclinical prion disease, and Hbb has been found to interact with subunits of ATP synthase in a study of Multiple Sclerosis (Xerxa et al., 2016, Brown et al., 2016).

Our work on the process of neurodegeneration has revealed mitochondrial dysfunction as an early event (Chakrabarti et al., 2010). We want to know how the mitochondrial milieu is adjusted during the period of early neural decline when symptoms are just becoming apparent. In order to do this we have utilised a classic mouse model of neurodegeneration, the *pcd*^*5j*^ mouse, and examined its mitochondrial proteome (Chakrabarti et al., 2006). The *pcd*^*5j*^ mouse undergoes a spontaneous degenerative process just after weaning. In fact, within 30 days of weaning, the cerebellum of this strain is nearly devoid of Purkinje Cells. The degeneration is cell specific and autonomous and is associated with an increase in mitophagy in presymptomatic cerebellum. We showed that there are differences in the mitochondrial proteome in the *pcd* mouse when compared to controls. One of the proteins we identified as changed is haemoglobin. We demonstrated that mitochondrial haemoglobin levels change in post-mortem Parkinson's disease brain (Shephard et al., 2014).

The identification of haemoglobin in mammalian cells, other than erythrocytes, was initially ascribed to blood contamination. However, it has been demonstrated that haemoglobin is detectable in neurons in vitro after the accepted lifespan of cultured erythrocytes has expired (Carlson et al., 2008). A growing body of evidence now demonstrates that haemoglobin is present in a range of cell types, in many of these cases the effect of intracellular haemoglobin is postulated to be protective. It has been shown prior to our work that hypoxia upregulates haemoglobin in alveolar epithelial cell lines (Grek et al., 2011). α- and β-haemoglobin (HbA and HbB respectively) respond to H_2_O_2_ induced oxidative stress by increasing their levels in HEK293 and hepatic cell lines (Liu et al., 2011). Haemoglobin mRNA and protein have been detected in rat, mouse and human brain (Biagioli et al., 2009). Our localisation of haemoglobin to the mitochondrial compartment has now been confirmed as a protective mechanism in circulating leukocytes (Shephard et al., 2014, Brunyanszki et al., 2015). In neurons erythropoietin induction has now been shown to increase mitochondrial function with increasing intraneuronal haemoglobin, in this case a reversal of memory impairment is recorded (Horng et al., 2015). A study of the mitochondrial proteome in Multiple Sclerosis cortex also finds beta haemoglobin expression is altered in this neurodegenerative disease (Biagioli et al., 2009).

Some studies already correlate haemoglobin levels with Parkinson's disease, simultaneously commenting upon the brain iron accumulation in Parkinson's and other neurodegenerations (Abbott et al., 2012, Savica et al., 2009). Since the majority of iron in the body is haemoglobin derived there may be also some connection with observations that serum and brain iron levels change in Parkinson's disease (Pichler et al., 2013). Potential interactions between haemoglobin and neurodegeneration are supported by the association of a functional polymorphism in the haemoglobin binding protein haptoglobin which influences susceptibility to idiopathic Parkinson's disease (Costa-Mallen et al., 2008). Much of the fluctuation in haemoglobin levels could be attributed to observed, yet unexplained anaemia in older individuals (Gaskell et al., 2008).

This study set out to understand localisation of haemoglobin to mitochondria and examine the levels of mitochondrial haemoglobin in Parkinson's disease post-mortem brains. We want to understand whether levels of mitochondrial haemoglobin change in Parkinson's disease compared with controls. We chose to examine mitochondrial haemoglobin levels in the substantia nigra - as the region of the brain where neuronal loss is most evident. We sampled cerebellar mitochondria for motor coordination related changes and also the cortex since it is least frequently associated with Parkinsonism.

## 2 Materials and methods

### 2.1 Human tissues

Human brain sections and frozen brain samples were obtained from, Human Tissue Authority approved, Nottingham Health Science Biobank (Nottingham University Hospitals NHS Trust) and Parkinson's UK Brain Bank (Imperial College London). The tissue banks granted us use of the tissue as end users. The Parkinson's tissue bank has approval from the Research Ethics Committee for Wales ref. 08/MRE09/31 + 5. The tissue collection and procedures at the Nottingham University Hospitals Biobank have been ethically approved by the Greater Manchester National Research Ethics Service. This study was granted specific ethics approval by both of the ethics committees serving the biobanks and also by the local ethics committee at the School of Veterinary Medicine and Science at the University of Nottingham. Tissues from human cerebellum, cortex and substantia nigra were used for mitochondrial isolations and were frozen at the time of post mortem (PM). Material used for immunohistochemistry was fixed in PFA at the time of PM. PM delay varied from a minimum of 2 h after death to a maximum of 6 days. Diagnoses of Parkinson's disease were confirmed at PM. Age at death varied from 58 to 87 years for Parkinson's disease brains, non-degenerative human control brains were age matched to within three years of Parkinson's disease samples, see Table 1. Early cases of Parkinson's are selected Braak 3 or 4, late cases are all Braak 5 and 6. Young onset cases are also Braak 5 or 6. Braak staging was performed according to published classification (Alafuzoff et al., 2009).

Ethical permission for the study was obtained through the brain banks from which the tissue was obtained. The entire study was also reviewed by our local ethics board.

### 2.2 Mitochondrial isolation

Mitochondria were prepared as previously described (Shephard et al., 2014). The quality of the crude fractions was confirmed using standard Western blotting techniques with nuclear, mitochondrial and cytoplasmic markers (Histone H3, ab 1791 (Abcam); COX IV ab16056 (Abcam); and HSP-90 ab13495 (Abcam) respectively). Sub-fractions were confirmed using outer membrane, inner membrane and inter-membrane space markers.

### 2.3 Immunoblotting

Western blotting was conducted as previously described (Shephard et al., 2014). Primary antibodies used were: Hba sc-21005 (Santa Cruz) 1:1000; and ab102758 (Abcam) 1:500 – for fly hypoxia; Hbb sc-22718 (Santa Cruz) 1:1000; COXIV ab16056 (Abcam) 1:1000; beta-actin ab8227 (Abcam) 1:4000; HSP-90 ab13495 (Abcam) 1:500; VDAC/Porin ab15895 (Abcam) 1:2000; NDUFS3 ab110246 (Abcam) 1:1000; SMAC/Diablo ab8115 (Abcam) 1:1000; dilution in 3% (*w*/*v*) BSA in TBS-T. Band densities were measured using Image J and samples were normalised to beta-actin. Using the normalised values the ratio of mitochondrial/cytoplasmic HbA and HbB were calculated. Data were analysed using the R statistical package http://www.r-project.org/ see supplemental data for script used to generate Fig. 3.

### 2.4 Hypoxic treatment

*Drosophila melanogaster* were maintained using standard techniques. Mixed populations of approx. 100 wild type flies were subjected to the following hypoxic conditions: 2.5% O_2_ 30 min 25 °C followed by normoxia 30 min 25 °C or 2.5% O_2_ 30 min 25 °C followed by normoxia 30 min 25 °C × 2. Mitochondria were isolated as described above and subjected to immunoblotting.

#### 2.4.1 Immunohistochemistry

Immunohistochemistry was performed as previously described (Shephard et al., 2014). Slides were visualised using a Pannoramic P250 scanner (3D Histech) with Lumencor Spectra Light Engine illumination source. Images were captured using a PCO Edge 5MP sCMOS monochrome camera and Carl Zeiss Plan ApoChromat 20x/0.8NA lens. The filter configuration used is shown in Supplementary Table 4.

## 3 Results

### 3.1 Mitochondrial HbA migrates from the intermembrane space to the outer membrane in affected male cerebellum

**Fig. 1:**
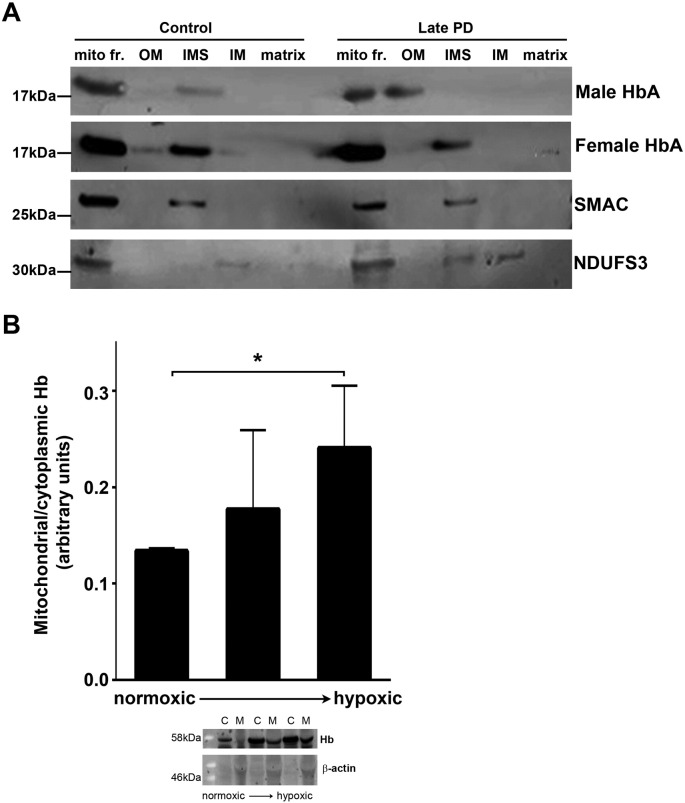
A. Mitochondrial HbA migrates from the intermembrane space to the outer membrane in affected human male cerebellum. Mitochondrial samples were sub-fractioned to allow examination of HbA localisation within the organelle, a representative gel is shown for each gender and control (total n = 8). The male Parkinson's disease brain demonstrated a shift in HbA localisation from the intermembrane space to the outer membrane fraction. This was not seen in the control or female Parkinson's brain mitochondria. Levels of HbA in the IMS were quantified in control and PD samples for both male and female patients (n = 2 for each), using Image J. Please see Supplemental Fig. 7 for all gel images. Levels of HbA in the IMS were significantly decreased in male PD compared with male control (p = 0.028 using unpaired two-tailed *t*-test). No significant change in female PD compared with female control (p $_amp_$gt; 0.05, unpaired two-tailed *t*-test). Mito fr – mitochondrial fraction, OM – outer membrane, IMS – inter membrane space, IM – inner membrane, M - matrix. B. Cycles of hypoxia result in increased hb in *Drosophila* mitochondrial fractions. Mitochondrial/cytoplasmic Hb levels determined using Western blotting, normalised to beta-actin. Hypoxia conditions: 2.5% O_2_ 30 min 25 °C followed by normoxia 30 min 25 °C (middle bar) 2.5% O_2_ 30 min 25 °C followed by normoxia 30 min 25 °C × 2 (right hand bar). 40–100 flies per condition. n = 3, * p $_amp_$lt; 0.05 (1 tailed *t*-test).

Sub-mitochondrial fractions were prepared from cerebella and interrogated for HbA content (Fig. 1A). Fractions were verified using appropriate antibodies SMAC and NDUFS3 for intermembrane space and inner membrane respectively. Control (male 80 years) and affected (male, 82 years, 18 years of disease) cerebella mitochondria were prepared and subfractionated. A female (85 years old, 18 years of disease) sub-fractionated sample is included for comparison. HbA content of the fractions was determined by western blotting. We found HbA to be present in the inter membrane space of the control sample which corroborates earlier findings (Shephard et al., 2014). In the affected sample there is little evidence of HbA in the inter membrane space. The HbA in the affected sample appears in the outer membrane fraction. Though the amount of HbA was not absolutely quantified, relative ratios suggest that there is a substantial quantity of this protein in or associated with the outer membrane of the affected sample mitochondria.

### 3.2 Hb levels increase in *Drosophila* mitochondrial fractions in response to cyclical hypoxia

In order to demonstrate an in vivo response of haemoglobin regulation in mitochondria we exposed fruit flies to cycles of hypoxia. Flies were in the group normoxic if they were not exposed to any hypoxic events. We tested two groups where the flies were exposed to two or three cycles of hypoxia (30 min at 2.5% oxygen), each followed by a recovery period (30 min) in normoxia. Mitochondria were prepared from whole flies in each of the three groups and Hb content ascertained in both cytoplasmic and mitochondrial fractions (Fig. 1B, Supplementary 1). The ratios of Hb content were plotted to reveal a significant increase (p < 0.05) in mitochondrial samples undergoing three rounds of hypoxia. Samples undergoing two rounds of hypoxia fitted with a trend towards increased mitochondrial Hb with hypoxia.

### 3.3 Mitochondrial versus cytoplasmic HbA and HbB ratios in early onset, early stage and late stage Parkinson's

**Fig. 2:**
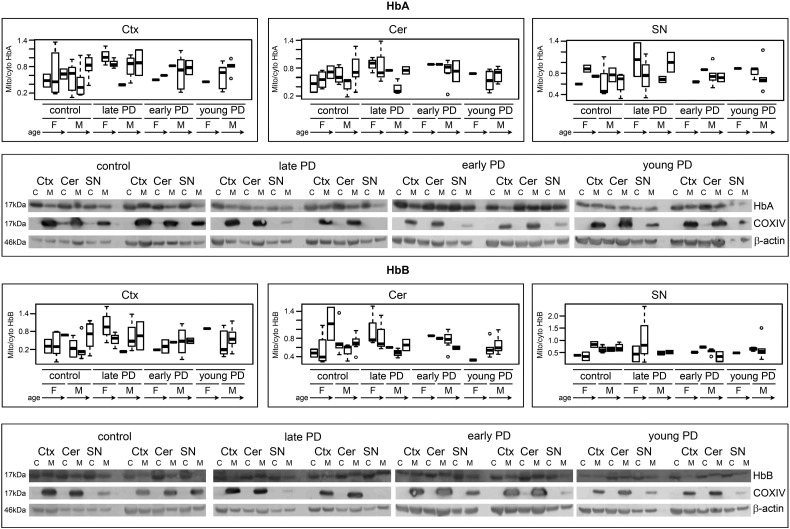
Representative gel images for mitochondrial/cytoplasmic Hb ratios - normalised to beta actin, were determined by Western blotting. COXIV antibody was utilised for quality control of mitochondrial versus cytoplasmic fractions. To summarise data, samples were grouped into age ranges (1 $_amp_$lt; 70, 2 70–79, 3 ≥ 80) and separated by sex and diagnosis type (control, late PD: late stage disease diagnosed over the age of 60, early PD: early stage disease diagnosed over the age of 60, young PD: early onset disease diagnosed under the age of 60). Values were visualised as boxplots. Boxplots show the median (line), interquartile range (box) and whiskers extend to 1.5 × the Inter quartile range. Extreme values beyond the whiskers are shown as circles. Ctx – Cortex, Cer – Cerebellum, SN – Substantia nigra. C M - cytoplasmic and mitochondrial fractions extracted from the same sample. Mito/cyto – ratio of mitochondrial Hb compared with cytoplasmic Hb. F-female, M – male. Full densitometry dataset is provided in Supplementary Table 2.

Data for calculating alpha and beta globin ratios were collected by western blot. Fractionated cell extracts were produced and run to give cytoplasmic versus mitochondrial ratios (Fig. 2). Ratios were calculated with reference to beta actin levels in the same lane. COXIV antibody indicated enrichment of mitochondrial fractions. Cortex, cerebellum and substantia nigra regions of the brain were examined for each sample. Using the arbitrary grouping of early Parkinson's (within 10 years of onset), late Parkinson's (beyond 10 years of disease) and young Parkinson's (onset before 60 years) we were unable to detect any major differences in mitochondrial/cytoplasmic ratios.

### 3.4 Mitochondrial haemoglobin ratios exhibit a dynamic range with disease duration

**Fig. 3:**
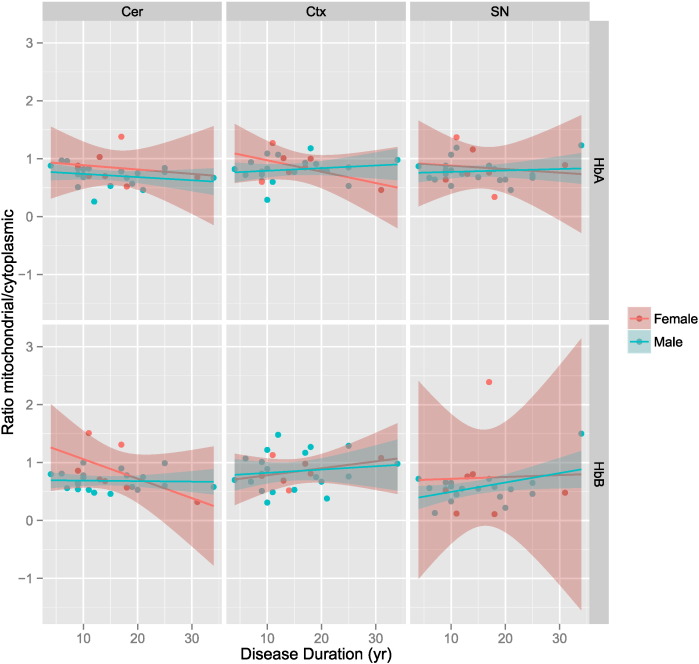
Scatterplots of mitochondrial/cytoplasmic Hb ratio versus disease duration in years. Scatterplots of the ratio of mitochondrial/cytoplasmic heamoglobin A and B in three tissues. Patient samples are split by sex and a linear model used to fit a trendline. The shaded areas show the 95% confidence limits of the fitted line. Calculations and plotting was conducted using ggplot in R (see supplemental R script which was used for generating figures). Orange circles (female), blue circles (male). Yr – years. Ctx – Cortex, Cer – Cerebellum, SN – Substantia nigra.

We re-examined our data set of mitochondrial/cytoplasmic ratios of alpha and beta haemoglobin, this time against disease duration. Specific immunoblotting of HbA and HbB were verified by LC-MSMS of trypsin digested proteins from immunopositive and negative gel bands (data provided in Supplementary Fig. 2). Scatter plots were generated from the whole set of immunoblotting data and show highly variable relationships as one might expect from post-mortem unrelated human brains with a sporadic disease (Fig. 3). However, two regions of the brain describe a trend towards a change when mitochondrial haemoglobin is measured. In the cortex, the gender specific scatter plots can be summarised by lines demonstrating an increase in mitochondrial HbA as the disease duration increases, this is most marked in the female brain tissues examined (red line). In the cerebellum mitochondrial HbB shows little dynamic change in mitochondrial HbB in male samples (blue line). The cerebellum was subsequently interrogated more carefully by sub-fractionation of the mitochondrial sample (Fig. 1A). In female samples mitochondrial HbB trends towards a decrease with disease duration going from a ratio of up to 1.5 at 10 years duration and then decreasing to a ratio ~ 0.3 by 30 years of disease course. Interestingly mitochondrial HbB ratio in substantia nigra indicates mostly low levels of this protein. This may reflect the proportion of neuron loss in this area of the brain and suggest that the losses in this area occur early in the disease process.

In order to account for any protein changes with regard to post mortem interval (PMI) we plotted HbB levels and also the mitochondrial marker COXIV against the delay after death in hours (Supplementary Fig. 3, Supplementary Fig. 4). These confirm that the levels of HbB we see against disease duration is not an effect of tissue changes after death has occurred. Brain banks usually have fewer female Parkinson's disease brains in their collections. We have analysed the full dataset we obtained but in order to be sure that differences in the numbers of females and males analysed did not skew our dataset we disease stage and age matched each female Parkinson's brain mitochondrial fraction (n = 7) with a male brain (n = 7) mitochondrial fraction and re-ran the data to show the gender difference is upheld with equal numbers of samples from each gender (Supplementary Fig. 6). Multiple linear regression analysis (software Genstat) was performed to verify that PMI is not a confounding factor in our analyses with disease duration – Supplementary Table 3.

### 3.5 The cellular distribution of HbB changes in female cerebellum with disease duration

**Fig. 4:**
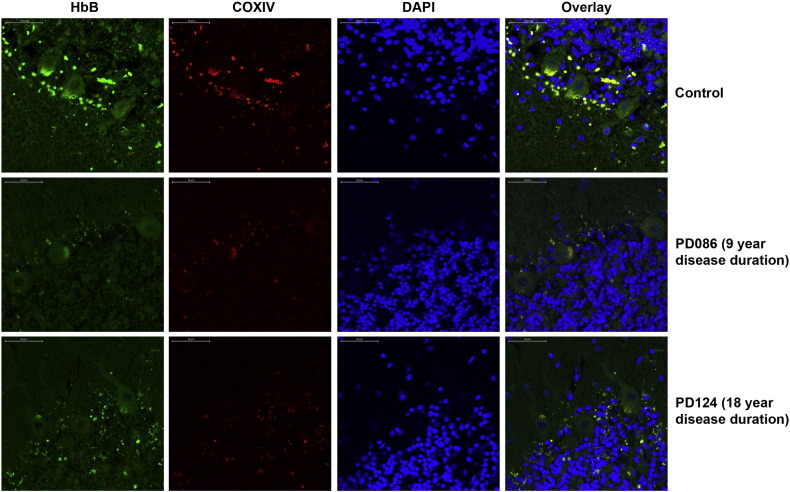
The cellular distribution of HbB appears changed in female cerebellum with disease duration. HbB and COXIV antibodies were utilised to visualise the physical location of mitochondria and HbB in the cerebellum of female individuals with different disease durations. It appears that with longer disease duration the overall quantity of COXIV is reduced and the location of HbB shifts in the longest disease course from in and around Purkinje cells observed in controls, to the granule cell layer in sections from 18 years disease duration. DAPI is used to stain nuclei. Filter sets used are detailed in the methodology.

Using antibodies to HbB and COXIV (for mitochondria) we localised mitochondrial haemoglobin in the female cerebellum (Fig. 4). In the female control cerebellum large Purkinje cells are labelled with both antibodies demonstrating the presence of mitochondrial HbB in the cell body. The cerebellum examined from a female patient 9 years into the disease suggests an overall decrease of mitochondrial HbB and mitochondria too. The patient shown with disease duration of 18 years appears to show COXIV staining in a different pattern again through the cerebellum, moving away from the Purkinje cell layer. HbB staining is increasingly distant from the Purkinje cell layer region of the cerebellum. Purkinje cell staining is relatively reduced. The brightness of the COXIV stain appears decreased in both affected brains. All the images presented were stained and imaged in a single batch to ensure valid qualitative comparison between images. The changes appear to be related to HbB protein localisation and this is something that needs to be looked into further.

## 4 Discussion

Our observations on the dynamic location of Hb led us to ask what could be the drivers of this proteins mobility within the cell and its organelles (Shephard et al., 2014). An obvious place to start is the manipulation of oxygen saturation and or delivery as has been done previously in vitro (Grek et al., 2011). We devised an experimental regime where we subject *Drosophila* to hypoxia. In order to emulate longer term fluctuations in oxygen availability we exposed flies to single or double hypoxic events, each followed by an equal period of recovery. Examination of mitochondrial fractions isolated from the flies revealed a significant increased ratio of mitochondrial Hb in response to multiple cycles of hypoxia and recovery. We suggest that this is indicative of a possible protective mechanism whereby Hb is sequestered to the mitochondrial organelle in conditions of hypoxia, perhaps in order to maintain an essential oxygen supply to the organelle. In long-lived brains this could occur in response to age related vascular changes when suboptimal quantities of oxygenated blood could be delivered to this most important organ (Qiu and Fratiglioni, 2015). It is possible that this is present at some level in multiple sclerosis where it is shown that HbB is found at higher levels in cortical neurons (Broadwater et al., 1812). However over a lifetime, unregulated sequestering of Hb in the organelle could lead to toxic levels within the intermembrane space. At the tipping point, overloaded organelles might be targeted for removal by mitophagy (Chakrabarti et al., 2009). In order to examine the localisation of Hb in human brains we carefully examined mitochondrial haemoglobin levels in control and age matched Parkinson's disease mitochondria.

We show that the levels of mitochondrial Hb trend towards a decrease in the cerebellum of females with Parkinson's. In males we find that the Hb content of the mitochondria does not appear decreased overall, sub-mitochondrial fractionation reveals that the Hb content though maintained, has moved into a different mitochondrial compartment. In fact the localisation in the outer membrane fraction does not allow us to specify whether Hb is merely associated with the external surface of the organelle or contained within the membrane organelle. Importantly, in males Hb is no longer detected in the inter membrane space where it is found in controls and in affected female cerebellum, this takes it away from the site of Complex 1 activity where it is shown to have an effect (Brunyanszki et al., 2015). Our sample set for this study is small, in particular there is some difficulty in obtaining large sample sets of female brains since the disease is less frequently encountered by women (Caslake et al., 2013). We would be interested to see whether other groups with similar or larger size sample sets can replicate these findings. Gender differences in Parkinson's disease are well documented, with females having a lower risk and a tremor dominant phenotype as well as reduced motor symptoms (Caslake et al., 2013). The cause of this is currently unknown but could be important in terms of generating neuroprotective therapies exploiting gender specific mechanisms. Our finding that mitochondrial Hb may be modulated differently according to gender could be a result of gender specific systemic availability of oxygen. Females in their reproductive years are particularly prone to anaemia (Pasricha et al., 2014). A recent article now connects haptoglobin a haemoglobin binding protein with low levels of serum iron, also with a greater effect when stratifying by gender (Costa-Mallen et al., 2015). There is gathering evidence that iron levels and anaemia are likely to be important players in the area of neurodegeneration, it is interesting to note that it is still unknown where the iron accumulation, seen for example in Parkinson's, originates Hametner et al., 2013, Mochizuki and Yasuda, 2012.

This study is important in identifying molecular changes in the cerebellum of PD post mortem brain. Recently the cerebellum has piqued the interest of PD researchers and has been postulated as a potential source of compensatory signalling (Lewis et al., 2013, Wu and Hallett, 2013, Rodríguez-Cueto et al., 2014). Change in glucose metabolism has been found to be an early event in the PD cerebellum and there have been a number of imaging studies that implicate the importance of this part of the brain in PD (Dunn et al., 2014). Our discovery of gender related molecular changes in PD mitochondria is an essential step in understanding the observed differences in symptomology between males and females with PD (Caslake et al., 2013, Colombo et al., 2015, Caranci et al., 2013, Solla et al., 2012, Cereda et al., 2014). As we are able to define the gender effect, common pathways can be better defined and compensatory loops, such as those proposed involving the cerebellum, can be targeted for therapy (Wu and Hallett, 2013).

The following are the supplementary data related to this article.

**Supplementary Fig. 1b:**
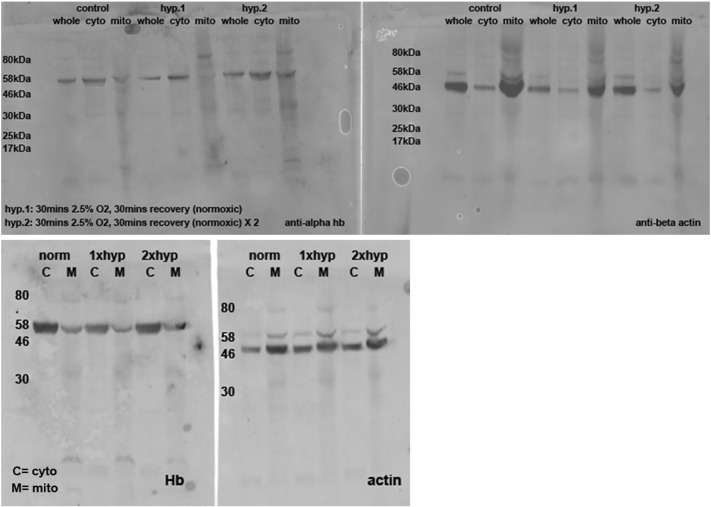
All western blots used to calculate fly Hb under cyclical hypoxia.

Supplementary Fig. 1 HbA antibody ab102758 (Abcam) gave a single band on gels with *Drosophila* mitochondrial fractions. The band size is ~ 56 kDa suggestive of a tetramer made up of units ~ 14 kDa. Drosophila haemoglobin has been identified relatively recently and established antibody based methodologies are not readily available, a putative Hb product is suggested to be about 17 kDa (Burmester et al., 2006). Using gradient gel based methodologies over a large size range we consider these values to approximate to the same molecular weight. We have included this Supplementary Fig. to demonstrate the lack of background reactivity of the antibody. Supplementary Fig. 1

Supplementary Fig. 2 Gel bands recognised by HbA and HbB antibodies used for detection in the human samples were excised. Samples were processed and trypsin digested using standard methods. LC-MSMS runs used a C18 reversed phase column, with a total run-time of 45 min. A mass exclude list was established from a blank gel piece (1E, which only contained some human keratins). All 4 western blot positive samples contain both HbB and HbA. A. shows an example of the positions on the protein sequence of the peptides matching HbB and HbA found in the mascot search for sample 1A_MP1914 (FEMALE PDC028 IMS 2/7/14). B. shows a list of all HbB and HbA peptide masses found by manual inspection in the mass spectrum for each of the 4 samples. Confirmation that the antibody used detects a protein identified as haemoglobin by mass spectrometry. Supplementary Fig. 2

**Supplementary Fig. 3:**
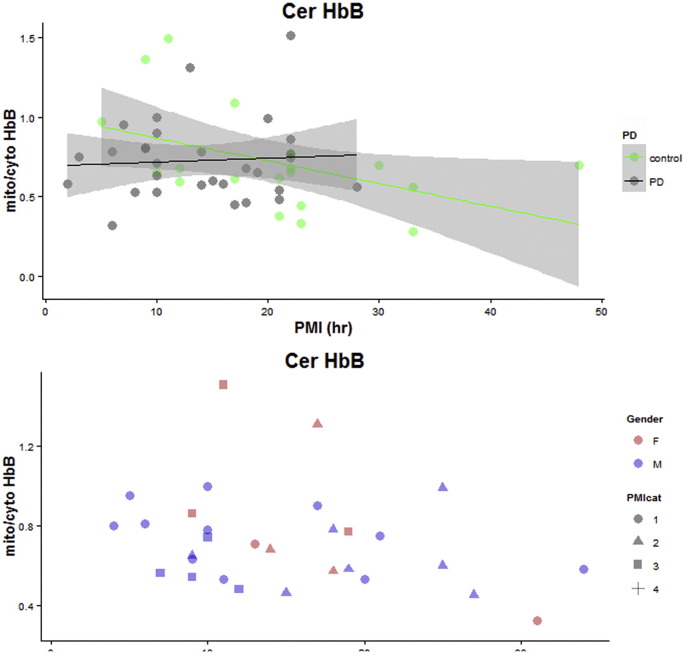
Ratio of mitochondrial versus cytoplasmic HbB in controls and Parkinson's cerebellum with increase in PMI. Control samples show a general decrease in HbB. The lower plot shows values for male and female HbB and is also coded for PMI category illustrating the lack of relationship between PMI and HbB ratios.

**Supplementary Fig. 4:**
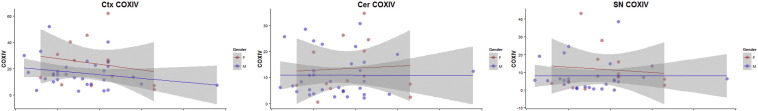
Levels of COXIV a mitochondrial marker, plotted against PMI. Supplementary Fig. 6. Equal numbers (n = 7 + 7) of male and female samples age-matched with similar disease duration also show a reduction in cerebellar HbB in the mitochondrial fraction from female cerebellum.

**Supplementary Fig. 5:**
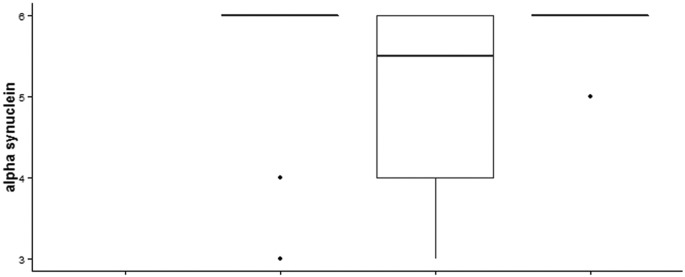
Alpha synuclein categories plotted for each group of Parkinson's brains.

**Supplementary Fig. 6:**
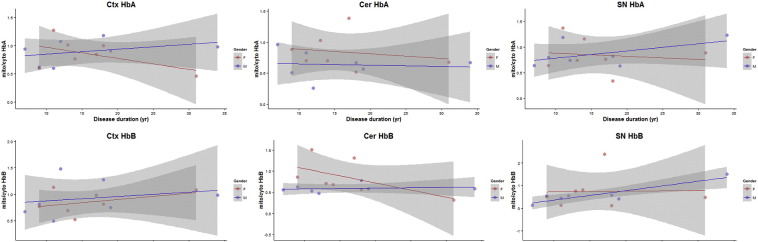
Haemoglobin ratios plotted for equal numbers of male and female age/disease matched brains.

**Supplemental Fig. 7:**
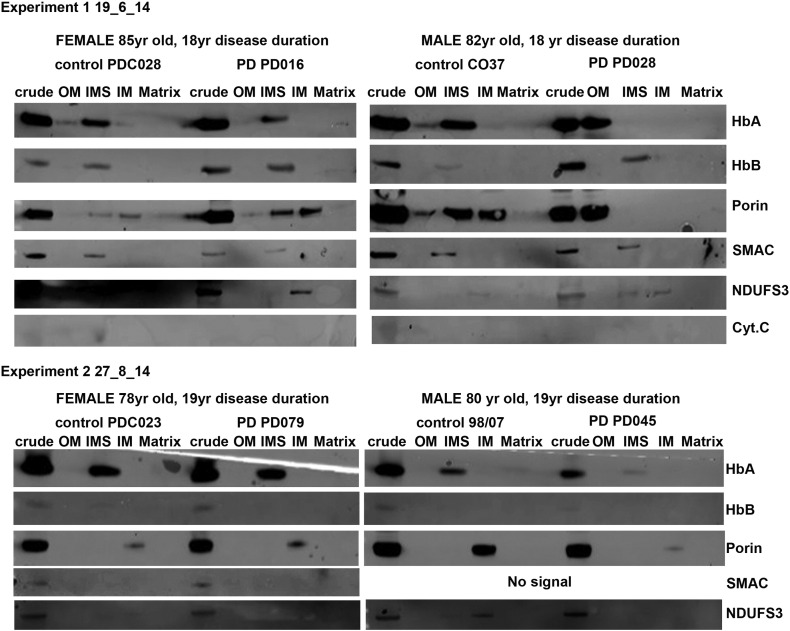

Supplementary R script Script coded in R used for generating Fig. 3 and supplementary scatter plots. Supplementary R script

Supplementary Table 1 Data for a search against only the human entries of the Swissprot database (SwissProt 2014_08) for sample 1A_MP1914 (FEMALE PDC028 IMS 2/7/14). Supplementary Table 1

Supplementary Table 2 Full western blot densitometry dataset. Supplementary Table 2

Supplementary Table 3 Accumulated analysis of variance. Sequential regression analysis was performed using Genstat, using the general linear regression tool, and adding the variables to the model in order with the largest mean square first. Supplementary Table 3

Supplementary Table 4 Slide scanner settings for immunofluorescence. Supplementary Table 4

Supplementary Table 5 Matched samples ‘equal numbers of male and female brains’, used to generate Supplementary Fig. 6. Supplementary Table 5

## Ethics

Human brain sections and frozen brain samples were obtained from, Human Tissue Authority approved, Nottingham Health Science Biobank (Nottingham University Hospitals NHS Trust) and Parkinson's UK Brain Bank (Imperial College London). The tissue banks granted us use of the tissue as end users. The Parkinson's tissue bank has approval from the Research Ethics Committee for Wales ref. 08/MRE09/31 + 5. The tissue collection and procedures at the Nottingham University Hospitals Biobank have been ethically approved by the Greater Manchester National Research Ethics Service. This study was granted specific ethical approval by both of the ethics committees serving the biobanks and also by the School of Veterinary Medicine and Science Local Ethics Committee at the University of Nottingham.

## Declarations of interest

The authors state that they have no competing interests associated with this work.

## Funding

This study was supported by a Michael J Fox Foundation ‘Rapid Response Innovation Award’. OGH is supported by the Biotechnology and Biological Sciences Research Council (GB) Doctoral Training Programme at the University of Nottingham and supplementary funding came from the University of Nottingham.

## Author contribution statement

FS, OGH, SL and LC conducted the research and analysed the data. RE analysed the data. LC and FS designed the study and obtained ethical approval and samples. LC and FS wrote the paper.

## Figures and Tables

**Table 1:**
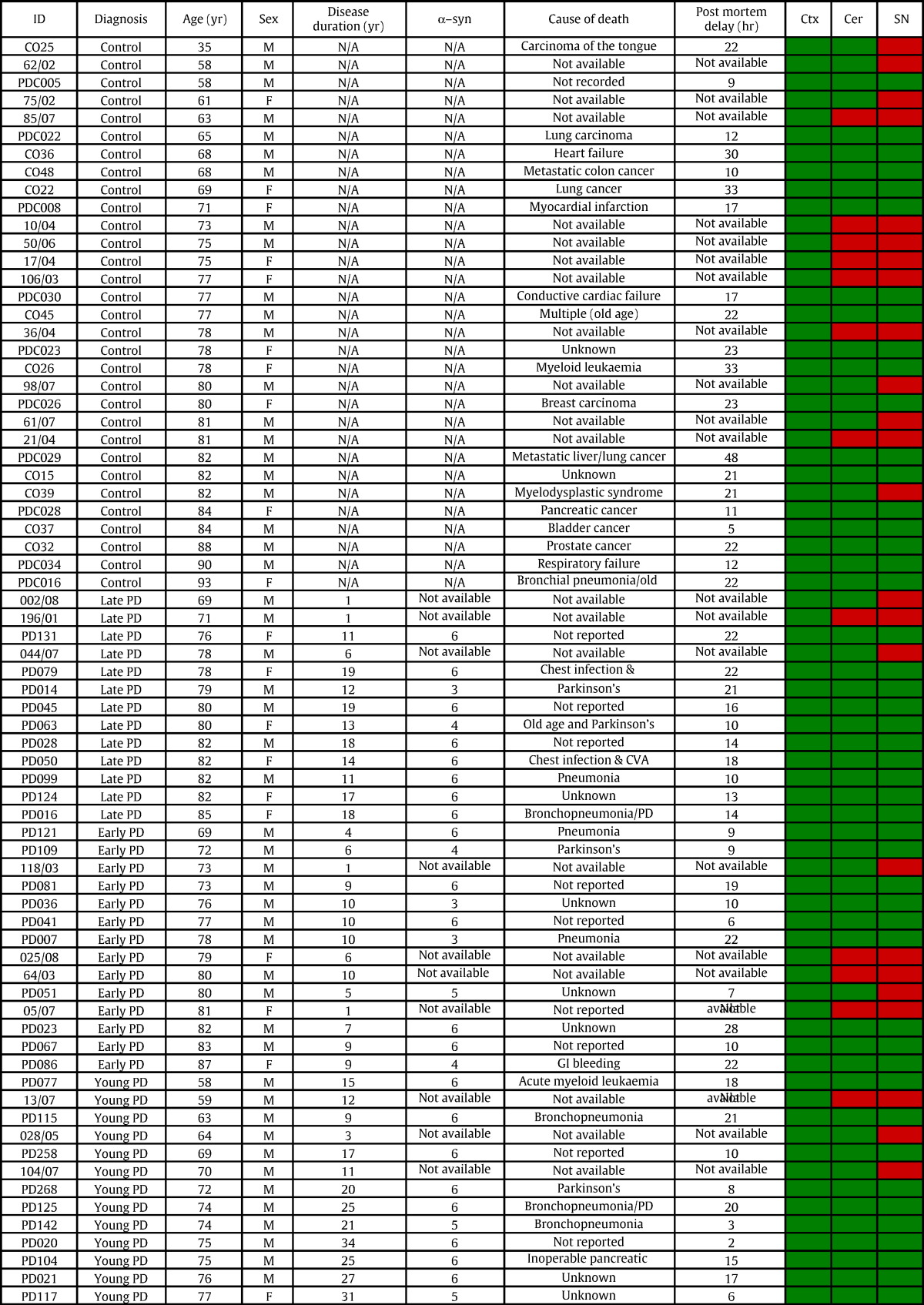
Parkinson's disease and matched control brain samples information. Post mortem interval (PMI) is stated in hours after death. Alpha – synuclein scoring by Parkinson's Brain Bank is included where available (not applicable NA, for control brains) the alpha-synuclein data for these tissues are visualised in Supplementary Fig. 5. PD1 is early onset cases before age of 60, PD2 is early disease with onset after 60 years, PD3 long disease duration onset after 60 available for this individual and the particular brain region, red boxes indicate the sample was not included. All tissues were obtained from Human Tissue Authority approved, Nottingham Health Science Biobank (Nottingham University Hospitals NHS Trust) and Parkinson's UK Brain Bank (Imperial College London).
