# Supplementary figures and images for: Analysis of Mitochondrial haemoglobin in Parkinson's disease brain
Source: Mitochondrion. 2016 Jul;29:45–52. doi: 10.1016/j.mito.2016.05.001 (PMC4940210; doi:10.1016/j.mito.2016.05.001)

### Supplementary Figure 1

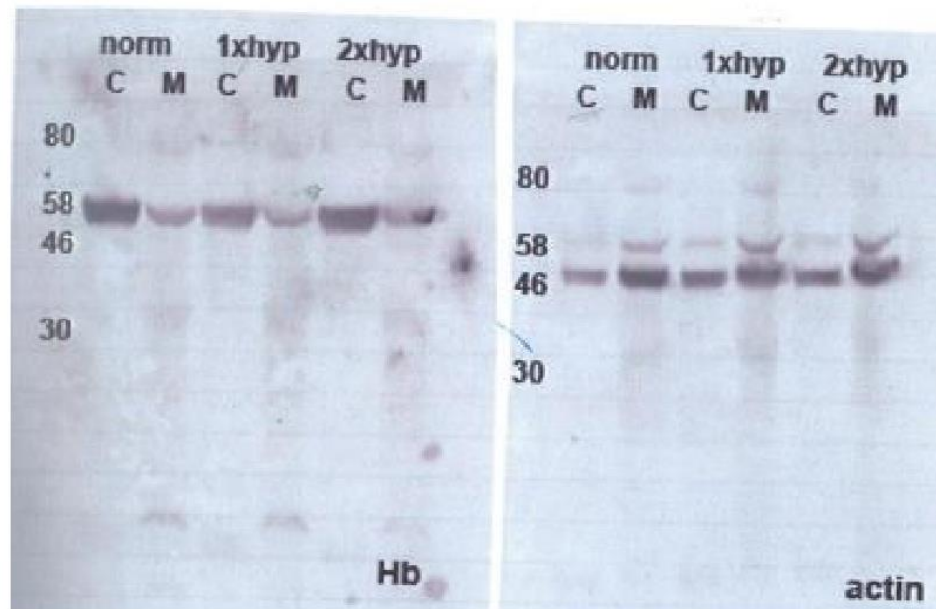

Supplement: Supplementary Fig. 1 — HbA antibody ab102758 (Abcam) gave a single band on gels with Drosophila mitochondrial fractions. The band size is ~ 56 kDa suggestive of a tetramer made up of units ~ 14 kDa. Drosophila haemoglobin has been identified relatively recently and established antibody based methodologies are not readily available, a putative Hb product is suggested to be about 17 kDa (Burmester et al., 2006). Using gradient gel based methodologies over a large size range we consider these values to approximate to the same molecular weight. We have included this Supplementary Fig. to demonstrate the lack of background reactivity of the antibody. [file mmc1.pdf]
