# Supplementary material for: Analysis of Mitochondrial haemoglobin in Parkinson's disease brain
Source: Mitochondrion. 2016 Jul;29:45–52. doi: 10.1016/j.mito.2016.05.001 (PMC4940210; doi:10.1016/j.mito.2016.05.001)
Supplement: Supplementary R script — Script coded in R used for generating Fig. 3 and supplementary scatter plots. [file mmc3.pdf]

```
#source("http://www.bioconductor.org/biocLite.R")
#install.packages("pvclust")
#install.packages("gplots")

library(gplots)
library(pvclust)
setwd("~/Documents/Collaborations/Chakrabarti_MitoMeth/Western.data/reanalysis.25.09.14")
data =
read.table("~/Documents/Collaborations/Chakrabarti_MitoMeth/Western.data/reanalysis.25.09.14/Chakrabarti.Parkinsons.data.txt",
header=TRUE, na.strings = "NA")
#
numbers <- data[6:11]
data.clean <-  as.matrix(na.omit(data))
numbers.clean <- na.omit(numbers)
t.numbers.clean <- (t(numbers.clean))
t.numbers <- (t(numbers))


#create 3 age categories
attach(data)
data$agecat[age > 80] <- "3"
data$agecat[age > 70 & age <= 80] <- "2"
data$agecat[age <= 70] <- "1"
detach(data)

#create PD/NOT category
attach(data)
data$PD[diagnosis=="control"] <- "0"
data$PD[diagnosis=="PD1"] <- "1"
data$PD[diagnosis=="PD2"] <- "1"
data$PD[diagnosis=="PD3"] <- "1"
detach(data)

pdf("Ce.age.sex.scatter.pdf")
par(mfrow = c(1,2))
plot(data$age[data$sex=="M"]~data$HbA.Ce[data$sex=="M"], pch = 20, col="blue", ylab ="Age (years)", xlab = "Ratio of
Mt:Cytoplasmic", main ="Ce (blue = male, red = female)")
points(data$age[data$sex=="F"]~data$HbA.Ce[data$sex=="F"], pch = 20, col="red", ylab ="Age (years)", xlab = "Ratio of
Mt:Cytoplasmic")
fit1 <- glm(data$age[data$sex=="M"] ~ data$HbA.Ce[data$sex=="M"])
co1 <- coef(fit1)
abline(fit1, col="blue", lwd=2)
fit2 <- glm(data$age[data$sex=="F"] ~ data$HbA.Ce[data$sex=="F"])
co1 <- coef(fit2)
abline(fit2, col="red", lwd=2)

plot(data$age[data$sex=="M"]~data$HbB.Ce[data$sex=="M"], pch = 20, col="blue", ylab ="Age (years)", xlab = "Ratio of
Mt:Cytoplasmic", main = "Ce (blue = male, red = female)")
points(data$age[data$sex=="F"]~data$HbB.Ce[data$sex=="F"], pch = 20, col="red", ylab ="Age (years)", xlab = "Ratio of
Mt:Cytoplasmic")
fit3 <- glm(data$age[data$sex=="M"] ~ data$HbB.Ce[data$sex=="M"])
co1 <- coef(fit3)
abline(fit3, col="blue", lwd=2)
fit4 <- glm(data$age[data$sex=="F"] ~ data$HbB.Ce[data$sex=="F"])
co1 <- coef(fit4)
abline(fit4, col="red", lwd=2)
dev.off()

pdf("FL.age.sex.scatter.pdf")
par(mfrow = c(1,2))
plot(data$age[data$sex=="M"]~data$HbA.FL[data$sex=="M"], pch = 20, col="blue", ylab ="Age (years)", xlab = "Ratio of
Mt:Cytoplasmic", main ="FL (blue = male, red = female)")
points(data$age[data$sex=="F"]~data$HbA.FL[data$sex=="F"], pch = 20, col="red", ylab ="Age (years)", xlab = "Ratio of
Mt:Cytoplasmic")
fit1 <- glm(data$age[data$sex=="M"] ~ data$HbA.FL[data$sex=="M"])
co1 <- coef(fit1)
abline(fit1, col="blue", lwd=2)
fit2 <- glm(data$age[data$sex=="F"] ~ data$HbA.FL[data$sex=="F"])
co1 <- coef(fit2)
abline(fit2, col="red", lwd=2)

plot(data$age[data$sex=="M"]~data$HbB.FL[data$sex=="M"], pch = 20, col="blue", ylab ="Age (years)", xlab = "Ratio of
Mt:Cytoplasmic", main = "FL (blue = male, red = female)")
points(data$age[data$sex=="F"]~data$HbB.FL[data$sex=="F"], pch = 20, col="red", ylab ="Age (years)", xlab = "Ratio of
Mt:Cytoplasmic")
fit3 <- glm(data$age[data$sex=="M"] ~ data$HbB.FL[data$sex=="M"])
co1 <- coef(fit3)
abline(fit3, col="blue", lwd=2)
fit4 <- glm(data$age[data$sex=="F"] ~ data$HbB.FL[data$sex=="F"])
co1 <- coef(fit4)
abline(fit4, col="red", lwd=2)
dev.off()

pdf("SN.age.sex.scatter.pdf")
par(mfrow = c(1,2))
plot(data$age[data$sex=="M"]~data$HbA.SN[data$sex=="M"], pch = 20, col="blue", ylab ="Age (years)", xlab = "Ratio of
Mt:Cytoplasmic", main ="SN (blue = male, red = female)")
points(data$age[data$sex=="F"]~data$HbA.SN[data$sex=="F"], pch = 20, col="red", ylab ="Age (years)", xlab = "Ratio of
Mt:Cytoplasmic")
fit1 <- glm(data$age[data$sex=="M"] ~ data$HbA.SN[data$sex=="M"])
co1 <- coef(fit1)
```

```
abline(fit1, col="blue", lwd=2)
fit2 <- glm(data$age[data$sex=="F"] ~ data$HbA.SN[data$sex=="F"])
co1 <- coef(fit2)
abline(fit2, col="red", lwd=2)

plot(data$age[data$sex=="M"]~data$HbB.SN[data$sex=="M"], pch = 20, col="blue", ylab ="Age (years)", xlab = "Ratio of
Mt:Cytoplasmic", main = "SN (blue = male, red = female)")
points(data$age[data$sex=="F"]~data$HbB.SN[data$sex=="F"], pch = 20, col="red", ylab ="Age (years)", xlab = "Ratio of
Mt:Cytoplasmic")
fit3 <- glm(data$age[data$sex=="M"] ~ data$HbB.SN[data$sex=="M"])
co1 <- coef(fit3)
abline(fit3, col="blue", lwd=2)
fit4 <- glm(data$age[data$sex=="F"] ~ data$HbB.SN[data$sex=="F"])
co1 <- coef(fit4)
abline(fit4, col="red", lwd=2)
dev.off()

h <- hclust(dist(numbers,method="euclidean"), method="complete")
pdf("clusters.pdf")
par(mfrow = c(2,2))
plot(h, labels=data$diagnosis, cex=0.5, main=NULL)
plot(h, labels=data$sex, cex=0.5, main=NULL)
plot(h, labels=data$age, cex=0.5, main=NULL)
plot(h, labels=data$agecat, cex=0.5, main=NULL)
dev.off()


par(mfrow = c(1,2))
boxplot(data$age[data$sex=="M"]~data$PD[data$sex=="M"], ylim =c(30,100))
boxplot(data$age[data$sex=="F"]~data$PD[data$sex=="F"], ylim =c(30,100))




pdf("clusters.clean.pdf")
result <- pvclust(numbers.clean, method.dist="euclidean", method.hclust="complete", nboot=1000)
plot(result)
dev.off()








sex.col <- c("red", "blue")
age.cat.col <- c("red", "blue", "darkgreen")
diagnosis.cat.col <- c("red", "blue", "darkgreen", "lightblue")

pdf("heatmap.sex.pdf")
heatmap.2(as.matrix(numbers), col=redgreen(75), scale="row", key=T, keysize=1,
          density.info="none", trace="none", cexCol=0.9,
          labRow=data$diagnosis,
          RowSideColors=sex.col[as.integer(data$sex)]
)
dev.off()

pdf("heatmap.age.pdf")
heatmap.2(as.matrix(numbers), col=redgreen(75), scale="row", key=T, keysize=1,
          density.info="none", trace="none", cexCol=0.9,
          labRow=data$diagnosis,
          RowSideColors=age.cat.col[as.integer(data$agecat)]
)
dev.off()

pdf("heatmap.diagnosis.pdf")
heatmap.2(as.matrix(numbers), col=redgreen(75), scale="row", key=T, keysize=1,
          density.info="none", trace="none", cexCol=0.9,
          labRow=data$diagnosis,
          RowSideColors=diagnosis.cat.col[as.integer(data$diagnosis)]
)
dev.off()


data.long <- reshape(data,
                     varying = c("HbA.FL","HbB.FL","HbA.Ce","HbB.Ce","HbA.SN","HbB.SN"),
                     v.names = "value",
                     timevar = "test",
                     times = c("HbA.FL","HbB.FL","HbA.Ce","HbB.Ce","HbA.SN","HbB.SN"),
                     direction = "long"
)



pdf("combined.boxplot.diagnosis.pdf")
par(mar=c(8,4,4,2), mfrow = c(3,2)) # increase x-axis margin.
boxplot(data.long$value[data.long$test=="HbA.FL"] ~
data.long$agecat[data.long$test=="HbA.FL"]*data.long$sex[data.long$test=="HbA.FL"]*data.long$diagnosis[data.long$test=="HbA.FL"],
main="Hb A FL", las=2)
boxplot(data.long$value[data.long$test=="HbB.FL"] ~
data.long$agecat[data.long$test=="HbB.FL"]*data.long$sex[data.long$test=="HbB.FL"]*data.long$diagnosis[data.long$test=="HbB.FL"],
```

```
main="Hb B FL", las=2)
boxplot(data.long$value[data.long$test=="HbA.Ce"] ~
data.long$agecat[data.long$test=="HbA.Ce"]*data.long$sex[data.long$test=="HbA.Ce"]*data.long$diagnosis[data.long$test=="HbA.Ce"],
main="Hb A Ce", las=2)
boxplot(data.long$value[data.long$test=="HbB.Ce"] ~
data.long$agecat[data.long$test=="HbB.Ce"]*data.long$sex[data.long$test=="HbB.Ce"]*data.long$diagnosis[data.long$test=="HbB.Ce"],
main="Hb B Ce", las=2)
boxplot(data.long$value[data.long$test=="HbA.SN"] ~
data.long$agecat[data.long$test=="HbA.SN"]*data.long$sex[data.long$test=="HbA.SN"]*data.long$diagnosis[data.long$test=="HbA.SN"],
main="Hb A SN", las=2)
boxplot(data.long$value[data.long$test=="HbB.SN"] ~
data.long$agecat[data.long$test=="HbB.SN"]*data.long$sex[data.long$test=="HbB.SN"]*data.long$diagnosis[data.long$test=="HbB.SN"],
main="Hb B SN", las=2)
dev.off()

pdf("combined.boxplot.age.pdf")
par(mar=c(8,4,4,2), mfrow = c(3,2)) # increase x-axis margin.
boxplot(data.long$value[data.long$test=="HbA.FL"] ~
data.long$diagnosis[data.long$test=="HbA.FL"]*data.long$sex[data.long$test=="HbA.FL"]*data.long$agecat[data.long$test=="HbA.FL"],
main="Hb A FL", las=2)
boxplot(data.long$value[data.long$test=="HbB.FL"] ~
data.long$diagnosis[data.long$test=="HbB.FL"]*data.long$sex[data.long$test=="HbB.FL"]*data.long$agecat[data.long$test=="HbB.FL"],
main="Hb B FL", las=2)
boxplot(data.long$value[data.long$test=="HbA.Ce"] ~
data.long$diagnosis[data.long$test=="HbA.Ce"]*data.long$sex[data.long$test=="HbA.Ce"]*data.long$agecat[data.long$test=="HbA.Ce"],
main="Hb A Ce", las=2)
boxplot(data.long$value[data.long$test=="HbB.Ce"] ~
data.long$diagnosis[data.long$test=="HbB.Ce"]*data.long$sex[data.long$test=="HbB.Ce"]*data.long$agecat[data.long$test=="HbB.Ce"],
main="Hb B Ce", las=2)
boxplot(data.long$value[data.long$test=="HbA.SN"] ~
data.long$diagnosis[data.long$test=="HbA.SN"]*data.long$sex[data.long$test=="HbA.SN"]*data.long$agecat[data.long$test=="HbA.SN"],
main="Hb A SN", las=2)
boxplot(data.long$value[data.long$test=="HbB.SN"] ~
data.long$diagnosis[data.long$test=="HbB.SN"]*data.long$sex[data.long$test=="HbB.SN"]*data.long$agecat[data.long$test=="HbB.SN"],
main="Hb B SN", las=2)
dev.off()

pdf("combined.boxplot.sex.pdf")
par(mar=c(8,4,4,2), mfrow = c(3,2)) # increase x-axis margin.
boxplot(data.long$value[data.long$test=="HbA.FL"] ~
data.long$diagnosis[data.long$test=="HbA.FL"]*data.long$agecat[data.long$test=="HbA.FL"]*data.long$sex[data.long$test=="HbA.FL"],
main="Hb A FL", las=2)
boxplot(data.long$value[data.long$test=="HbB.FL"] ~
data.long$diagnosis[data.long$test=="HbB.FL"]*data.long$agecat[data.long$test=="HbB.FL"]*data.long$sex[data.long$test=="HbB.FL"],
main="Hb B FL", las=2)
boxplot(data.long$value[data.long$test=="HbA.Ce"] ~
data.long$diagnosis[data.long$test=="HbA.Ce"]*data.long$agecat[data.long$test=="HbA.Ce"]*data.long$sex[data.long$test=="HbA.Ce"],
main="Hb A Ce", las=2)
boxplot(data.long$value[data.long$test=="HbB.Ce"] ~
data.long$diagnosis[data.long$test=="HbB.Ce"]*data.long$agecat[data.long$test=="HbB.Ce"]*data.long$sex[data.long$test=="HbB.Ce"],
main="Hb B Ce", las=2)
boxplot(data.long$value[data.long$test=="HbA.SN"] ~
data.long$diagnosis[data.long$test=="HbA.SN"]*data.long$agecat[data.long$test=="HbA.SN"]*data.long$sex[data.long$test=="HbA.SN"],
main="Hb A SN", las=2)
boxplot(data.long$value[data.long$test=="HbB.SN"] ~
data.long$diagnosis[data.long$test=="HbB.SN"]*data.long$agecat[data.long$test=="HbB.SN"]*data.long$sex[data.long$test=="HbB.SN"],
main="Hb B SN", las=2)
dev.off()


pdf("FL.sex.boxplot.pdf")
par(mfrow = c(2,2))
diagnosis.group.labels <- c("Control", "PD1", "PD2", "PD3")
boxplot(data$HbA.FL[data$sex=="M"] ~ data$diagnosis[data$sex=="M"],
        names=diagnosis.group.labels,
        col="lightblue",
        main = "HbA FL Male"
)
boxplot(data$HbA.FL[data$sex=="F"] ~ data$diagnosis[data$sex=="F"],
        names=diagnosis.group.labels,
        col="lightblue",
        main = "HbA FL Female"
)
boxplot(data$HbB.FL[data$sex=="M"] ~ data$diagnosis[data$sex=="M"],
        names=diagnosis.group.labels,
        col="lightblue",
        main = "HbB FL Male"
)
boxplot(data$HbB.FL[data$sex=="F"] ~ data$diagnosis[data$sex=="F"],
        names=diagnosis.group.labels,
        col="lightblue",
        main = "HbB FL Female"
)
dev.off()

duration.clean.Ce <- na.omit(subset(data, select=c("diagnosis","sex","disease.duration.years","HbA.Ce","HbB.Ce")))
duration.clean.FL <- na.omit(subset(data, select=c("diagnosis","sex","disease.duration.years","HbA.FL","HbB.FL")))
duration.clean.SN <- na.omit(subset(data, select=c("diagnosis","sex","disease.duration.years","HbA.SN","HbB.SN")))


# plot male and female on single plot with linear regression,
pdf("Ce.sex.disease.duration.scatter.pdf")
```

```
par(mfrow = c(1,2))
x = duration.clean.Ce$disease.duration.years[duration.clean.Ce$sex=="M"]
y = duration.clean.Ce$HbA.Ce[duration.clean.Ce$sex=="M"]
x1 = duration.clean.Ce$disease.duration.years[duration.clean.Ce$sex=="F"]
y1 = duration.clean.Ce$HbA.Ce[duration.clean.Ce$sex=="F"]

x2 = duration.clean.Ce$disease.duration.years[duration.clean.Ce$sex=="M"]
y2 = duration.clean.Ce$HbB.Ce[duration.clean.Ce$sex=="M"]
x3 = duration.clean.Ce$disease.duration.years[duration.clean.Ce$sex=="F"]
y3 = duration.clean.Ce$HbB.Ce[duration.clean.Ce$sex=="F"]

plot (y~x, col="blue", main = "Cerebellum HbA", xlab="Duration (years)", ylab="HbA", ylim = c(0,(max(y,y1,y2,y3)+0.1)),
pch=20)
fit <- lm(y~x)
abline(fit, col="blue", lwd=2)
points (y1~x1, col="red",pch=20)
fit1 <- lm(y1~x1)
abline(fit1, col="red", lwd=2)

plot (y2~x2, col="blue", main = "Cerebellum HbB", xlab="Duration (years)", ylab="HbB", ylim = c(0,(max(y,y1,y2,y3)+0.1)),
pch=20)
fit2 <- lm(y2~x2)
abline(fit, col="blue", lwd=2)
points (y3~x3, col="red",pch=20)
fit3 <- lm(y3~x3)
abline(fit3, col="red", lwd=2)
dev.off()

pdf("FL.sex.disease.duration.scatter.pdf")
par(mfrow = c(1,2))
x = duration.clean.FL$disease.duration.years[duration.clean.FL$sex=="M"]
y = duration.clean.FL$HbA.FL[duration.clean.FL$sex=="M"]
x1 = duration.clean.FL$disease.duration.years[duration.clean.FL$sex=="F"]
y1 = duration.clean.FL$HbA.FL[duration.clean.FL$sex=="F"]

x2 = duration.clean.FL$disease.duration.years[duration.clean.FL$sex=="M"]
y2 = duration.clean.FL$HbB.FL[duration.clean.FL$sex=="M"]
x3 = duration.clean.FL$disease.duration.years[duration.clean.FL$sex=="F"]
y3 = duration.clean.FL$HbB.FL[duration.clean.FL$sex=="F"]

plot (y~x, col="blue", main = "Frontal Lobe HbA", xlab="Duration (years)", ylab="HbA", ylim = c(0,
(max(y,y1,y2,y3)+0.1)),pch=20)
fit <- lm(y~x)
abline(fit, col="blue", lwd=2)
points (y1~x1, col="red",pch=20)
fit1 <- lm(y1~x1)
abline(fit1, col="red", lwd=2)

plot (y2~x2, col="blue", main = "Frontal Lobe HbB", xlab="Duration (years)", ylab="HbB", ylim = c(0,
(max(y,y1,y2,y3)+0.1)),pch=20)
fit2 <- lm(y2~x2)
abline(fit, col="blue", lwd=2)
points (y3~x3, col="red",pch=20)
fit3 <- lm(y3~x3)
abline(fit3, col="red", lwd=2)
dev.off()


pdf("SN.sex.disease.duration.scatter.pdf")
par(mfrow = c(1,2))
x = duration.clean.SN$disease.duration.years[duration.clean.SN$sex=="M"]
y = duration.clean.SN$HbA.SN[duration.clean.SN$sex=="M"]
x1 = duration.clean.SN$disease.duration.years[duration.clean.SN$sex=="F"]
y1 = duration.clean.SN$HbA.SN[duration.clean.SN$sex=="F"]

x2 = duration.clean.SN$disease.duration.years[duration.clean.SN$sex=="M"]
y2 = duration.clean.SN$HbB.SN[duration.clean.SN$sex=="M"]
x3 = duration.clean.SN$disease.duration.years[duration.clean.SN$sex=="F"]
y3 = duration.clean.SN$HbB.SN[duration.clean.SN$sex=="F"]

plot (y~x, col="blue", main = "Substantia Nigra HbA", xlab="Duration (years)", ylab="HbA", ylim = c(0,
(max(y,y1,y2,y3)+0.1)),pch=20)
fit <- lm(y~x)
abline(fit, col="blue", lwd=2)
points (y1~x1, col="red",pch=20)
fit1 <- lm(y1~x1)
abline(fit1, col="red", lwd=2)

plot (y2~x2, col="blue", main = "Substantia Nigra HbB", xlab="Duration (years)", ylab="HbB", ylim = c(0,
(max(y,y1,y2,y3)+0.1)),pch=20)
fit2 <- lm(y2~x2)
abline(fit, col="blue", lwd=2)
points (y3~x3, col="red",pch=20)
fit3 <- lm(y3~x3)
abline(fit3, col="red", lwd=2)
dev.off()


# ADD CONFIDENCE INTERVALS
pdf("Ce.sex.disease.duration.scatter.CI.pdf")
par(mfrow = c(2,2))
x = duration.clean.Ce$disease.duration.years[duration.clean.Ce$sex=="M"]
```

```
y = duration.clean.Ce$HbA.Ce[duration.clean.Ce$sex=="M"]
x1 = duration.clean.Ce$disease.duration.years[duration.clean.Ce$sex=="F"]
y1 = duration.clean.Ce$HbA.Ce[duration.clean.Ce$sex=="F"]

x2 = duration.clean.Ce$disease.duration.years[duration.clean.Ce$sex=="M"]
y2 = duration.clean.Ce$HbB.Ce[duration.clean.Ce$sex=="M"]
x3 = duration.clean.Ce$disease.duration.years[duration.clean.Ce$sex=="F"]
y3 = duration.clean.Ce$HbB.Ce[duration.clean.Ce$sex=="F"]

maxy = (max(y,y1,y2,y3) + 0.1)

plot(y~x, col= "blue", pch=20, main = "Ce Male", xlab="Duration (years)", ylab="HbA", ylim = c(0,maxy))
fit <- lm(y~x)
abline(fit, col="blue", lwd=2)
newx <- seq(min(x,x1,x2,x3), max(x,x1,x2,x3), 0.5)
prd<-predict(fit,newdata=data.frame(x=newx),interval = c("confidence"))
lines(newx,prd[,2],col="blue",lty=2)
lines(newx,prd[,3],col="blue",lty=2)

plot(y1~x1, col= "red", pch=20, main = "Ce Female", xlab="Duration (years)", ylab="HbA", ylim = c(0,maxy))
fit1 <- lm(y1~x1)
abline(fit1, col="red", lwd=2)
newx1 <- seq(min(x,x1,x2,x3), max(x,x1,x2,x3), 0.5)
prd1<-predict(fit1,newdata=data.frame(x1=newx1),interval = c("confidence"))
lines(newx1,prd1[,2],col="red",lty=2)
lines(newx1,prd1[,3],col="red",lty=2)

plot(y2~x2, col= "blue", pch=20, main = "Ce Male", xlab="Duration (years)", ylab="HbB", ylim = c(0,maxy))
fit2 <- lm(y2~x2)
abline(fit2, col="blue", lwd=2)
newx2 <- seq(min(x,x1,x2,x3), max(x,x1,x2,x3), 0.5)
prd2 <-predict(fit2,newdata=data.frame(x2=newx2),interval = c("confidence"))
lines(newx2,prd2[,2],col="blue",lty=2)
lines(newx2,prd2[,3],col="blue",lty=2)

plot(y3~x3, col= "red", pch=20, main = "Ce Female", xlab="Duration (years)", ylab="HbB", ylim = c(0,maxy))
fit3 <- lm(y3~x3)
abline(fit3, col="red", lwd=2)
newx3 <- seq(min(x,x1,x2,x3), max(x,x1,x2,x3), 0.5)
prd3 <-predict(fit3,newdata=data.frame(x3=newx3),interval = c("confidence"))
lines(newx3,prd3[,2],col="red",lty=2)
lines(newx3,prd3[,3],col="red",lty=2)
dev.off()


pdf("FL.sex.disease.duration.scatter.CI.pdf")
par(mfrow = c(2,2))
x = duration.clean.FL$disease.duration.years[duration.clean.FL$sex=="M"]
y = duration.clean.FL$HbA.FL[duration.clean.FL$sex=="M"]
x1 = duration.clean.FL$disease.duration.years[duration.clean.FL$sex=="F"]
y1 = duration.clean.FL$HbA.FL[duration.clean.FL$sex=="F"]

x2 = duration.clean.FL$disease.duration.years[duration.clean.FL$sex=="M"]
y2 = duration.clean.FL$HbB.FL[duration.clean.FL$sex=="M"]
x3 = duration.clean.FL$disease.duration.years[duration.clean.FL$sex=="F"]
y3 = duration.clean.FL$HbB.FL[duration.clean.FL$sex=="F"]

maxy = (max(y,y1,y2,y3) + 0.1)

plot(y~x, col= "blue", pch=20, main = "FL Male", xlab="Duration (years)", ylab="HbA", ylim = c(0,maxy))
fit <- lm(y~x)
abline(fit, col="blue", lwd=2)
newx <- seq(min(x,x1,x2,x3), max(x,x1,x2,x3), 0.5)
prd<-predict(fit,newdata=data.frame(x=newx),interval = c("confidence"))
lines(newx,prd[,2],col="blue",lty=2)
lines(newx,prd[,3],col="blue",lty=2)

plot(y1~x1, col= "red", pch=20, main = "FL Female", xlab="Duration (years)", ylab="HbA", ylim = c(0,maxy))
fit1 <- lm(y1~x1)
abline(fit1, col="red", lwd=2)
newx1 <- seq(min(x,x1,x2,x3), max(x,x1,x2,x3), 0.5)
prd1<-predict(fit1,newdata=data.frame(x1=newx1),interval = c("confidence"))
lines(newx1,prd1[,2],col="red",lty=2)
lines(newx1,prd1[,3],col="red",lty=2)

plot(y2~x2, col= "blue", pch=20, main = "FL Male", xlab="Duration (years)", ylab="HbB", ylim = c(0,maxy))
fit2 <- lm(y2~x2)
abline(fit2, col="blue", lwd=2)
newx2 <- seq(min(x,x1,x2,x3), max(x,x1,x2,x3), 0.5)
prd2 <-predict(fit2,newdata=data.frame(x2=newx2),interval = c("confidence"))
lines(newx2,prd2[,2],col="blue",lty=2)
lines(newx2,prd2[,3],col="blue",lty=2)

plot(y3~x3, col= "red", pch=20, main = "FL Female", xlab="Duration (years)", ylab="HbB", ylim = c(0,maxy))
fit3 <- lm(y3~x3)
abline(fit3, col="red", lwd=2)
newx3 <- seq(min(x,x1,x2,x3), max(x,x1,x2,x3), 0.5)
prd3 <-predict(fit3,newdata=data.frame(x3=newx3),interval = c("confidence"))
lines(newx3,prd3[,2],col="red",lty=2)
lines(newx3,prd3[,3],col="red",lty=2)
dev.off()
```

```
pdf("SN.sex.disease.duration.scatter.CI.pdf")
par(mfrow = c(2,2))
x = duration.clean.SN$disease.duration.years[duration.clean.SN$sex=="M"]
y = duration.clean.SN$HbA.SN[duration.clean.SN$sex=="M"]
x1 = duration.clean.SN$disease.duration.years[duration.clean.SN$sex=="F"]
y1 = duration.clean.SN$HbA.SN[duration.clean.SN$sex=="F"]

x2 = duration.clean.SN$disease.duration.years[duration.clean.SN$sex=="M"]
y2 = duration.clean.SN$HbB.SN[duration.clean.SN$sex=="M"]
x3 = duration.clean.SN$disease.duration.years[duration.clean.SN$sex=="F"]
y3 = duration.clean.SN$HbB.SN[duration.clean.SN$sex=="F"]

maxy = (max(y,y1,y2,y3) + 0.1)

plot(y~x, col= "blue", pch=20, main = "SN Male", xlab="Duration (years)", ylab="HbA", ylim = c(0,maxy))
fit <- lm(y~x)
abline(fit, col="blue", lwd=2)
newx <- seq(min(x,x1,x2,x3), max(x,x1,x2,x3), 0.5)
prd<-predict(fit,newdata=data.frame(x=newx),interval = c("confidence"))
lines(newx,prd[,2],col="blue",lty=2)
lines(newx,prd[,3],col="blue",lty=2)

plot(y1~x1, col= "red", pch=20, main = "SN Female", xlab="Duration (years)", ylab="HbA", ylim = c(0,maxy))
fit1 <- lm(y1~x1)
abline(fit1, col="red", lwd=2)
newx1 <- seq(min(x,x1,x2,x3), max(x,x1,x2,x3), 0.5)
prd1<-predict(fit1,newdata=data.frame(x1=newx1),interval = c("confidence"))
lines(newx1,prd1[,2],col="red",lty=2)
lines(newx1,prd1[,3],col="red",lty=2)

plot(y2~x2, col= "blue", pch=20, main = "SN Male", xlab="Duration (years)", ylab="HbB", ylim = c(0,maxy))
fit2 <- lm(y2~x2)
abline(fit2, col="blue", lwd=2)
newx2 <- seq(min(x,x1,x2,x3), max(x,x1,x2,x3), 0.5)
prd2 <-predict(fit2,newdata=data.frame(x2=newx2),interval = c("confidence"))
lines(newx2,prd2[,2],col="blue",lty=2)
lines(newx2,prd2[,3],col="blue",lty=2)

plot(y3~x3, col= "red", pch=20, main = "SN Female", xlab="Duration (years)", ylab="HbB", ylim = c(0,maxy))
fit3 <- lm(y3~x3)
abline(fit3, col="red", lwd=2)
newx3 <- seq(min(x,x1,x2,x3), max(x,x1,x2,x3), 0.5)
prd3 <-predict(fit3,newdata=data.frame(x3=newx3),interval = c("confidence"))
lines(newx3,prd3[,2],col="red",lty=2)
lines(newx3,prd3[,3],col="red",lty=2)
dev.off()








pdf("Ce.sex.boxplot.pdf")
par(mfrow = c(2,2))
diagnosis.group.labels <- c("Control", "PD1", "PD2", "PD3")
boxplot(data$HbA.Ce[data$sex=="M"] ~ data$diagnosis[data$sex=="M"],
        names=diagnosis.group.labels,
        col="lightblue",
        main = "HbA Ce Male"
)
boxplot(data$HbA.Ce[data$sex=="F"] ~ data$diagnosis[data$sex=="F"],
        names=diagnosis.group.labels,
        col="lightblue",
        main = "HbA Ce Female"
)
boxplot(data$HbB.Ce[data$sex=="M"] ~ data$diagnosis[data$sex=="M"],
        names=diagnosis.group.labels,
        col="lightblue",
        main = "HbB Ce Male"
)
boxplot(data$HbB.Ce[data$sex=="F"] ~ data$diagnosis[data$sex=="F"],
        names=diagnosis.group.labels,
        col="lightblue",
        main = "HbB Ce Female"
)
dev.off()


pdf("SN.sex.boxplot.pdf")
par(mfrow = c(2,2))
diagnosis.group.labels <- c("Control", "PD1", "PD2", "PD3")
boxplot(data$HbA.SN[data$sex=="M"] ~ data$diagnosis[data$sex=="M"],
        names=diagnosis.group.labels,
        col="lightblue",
        main = "HbA SN Male"
)
boxplot(data$HbA.SN[data$sex=="F"] ~ data$diagnosis[data$sex=="F"],
        names=diagnosis.group.labels,
        col="lightblue",
```

```
        main = "HbA SN Female"
)
boxplot(data$HbB.SN[data$sex=="M"] ~ data$diagnosis[data$sex=="M"],
        names=diagnosis.group.labels,
        col="lightblue",
        main = "HbB SN Male"
)
boxplot(data$HbB.SN[data$sex=="F"] ~ data$diagnosis[data$sex=="F"],
        names=diagnosis.group.labels,
        col="lightblue",
        main = "HbB SN Female"
)
dev.off()

pdf("histogram.age.pdf")
hist(data$age[data$sex=="M"])
hist(data$age[data$sex=="F"])
dev.off()


pdf("FL.age.boxplot.pdf")
par(mfrow = c(2,2))
age.group.labels <- c("<70", "70-79", "80+")

boxplot(data$HbA.FL[data$sex=="M"] ~ data$agecat[data$sex=="M"],
        names=age.group.labels,
        col="lightblue",
        xlab="Age (years)",
        main = "HbA FL Male"
)

boxplot(data$HbA.FL[data$sex=="F"] ~ data$agecat[data$sex=="F"],
        names=age.group.labels,
        col="lightblue",
        xlab="Age (years)",
        main = "HbA FL Female"
)

boxplot(data$HbB.FL[data$sex=="M"] ~ data$agecat[data$sex=="M"],
        names=age.group.labels,
        col="lightblue",
        xlab="Age (years)",
        main = "HbB FL Male"
)

boxplot(data$HbB.FL[data$sex=="F"] ~ data$agecat[data$sex=="F"],
        names=age.group.labels,
        col="lightblue",
        xlab="Age (years)",
        main = "HbB FL Female"
)
dev.off()


pdf("Ce.age.boxplot.pdf")
par(mfrow = c(2,2))
age.group.labels <- c("<70", "70-79", "80+")

boxplot(data$HbA.Ce[data$sex=="M"] ~ data$agecat[data$sex=="M"],
        names=age.group.labels,
        col="lightblue",
        xlab="Age (years)",
        main = "HbA Ce Male"
)

boxplot(data$HbA.Ce[data$sex=="F"] ~ data$agecat[data$sex=="F"],
        names=age.group.labels,
        col="lightblue",
        xlab="Age (years)",
        main = "HbA Ce Female"
)

boxplot(data$HbB.Ce[data$sex=="M"] ~ data$agecat[data$sex=="M"],
        names=age.group.labels,
        col="lightblue",
        xlab="Age (years)",
        main = "HbB Ce Male"
)

boxplot(data$HbB.Ce[data$sex=="F"] ~ data$agecat[data$sex=="F"],
        names=age.group.labels,
        col="lightblue",
        xlab="Age (years)",
        main = "HbB Ce Female"
)
dev.off()


pdf("SN.age.boxplot.pdf")
par(mfrow = c(2,2))
age.group.labels <- c("<70", "70-79", "80+")
```

```
boxplot(data$HbA.SN[data$sex=="M"] ~ data$agecat[data$sex=="M"],
        names=age.group.labels,
        col="lightblue",
        xlab="Age (years)",
        main = "HbA SN Male"
)

boxplot(data$HbA.SN[data$sex=="F"] ~ data$agecat[data$sex=="F"],
        names=age.group.labels,
        col="lightblue",
        xlab="Age (years)",
        main = "HbA SN Female"
)

boxplot(data$HbB.SN[data$sex=="M"] ~ data$agecat[data$sex=="M"],
        names=age.group.labels,
        col="lightblue",
        xlab="Age (years)",
        main = "HbB SN Male"
)

boxplot(data$HbB.SN[data$sex=="F"] ~ data$agecat[data$sex=="F"],
        names=age.group.labels,
        col="lightblue",
        xlab="Age (years)",
        main = "HbB SN Female"
)
dev.off()



########################################################################
# COMBINE CONFIDENCE
#########################################################################

# plot male and female on single plot with linear regression,and CI
pdf("Ce.sex.disease.duration.scatter.CI.bothsex.pdf")
par(mfrow = c(1,2))
x = duration.clean.Ce$disease.duration.years[duration.clean.Ce$sex=="M"]
y = duration.clean.Ce$HbA.Ce[duration.clean.Ce$sex=="M"]
x1 = duration.clean.Ce$disease.duration.years[duration.clean.Ce$sex=="F"]
y1 = duration.clean.Ce$HbA.Ce[duration.clean.Ce$sex=="F"]
x2 = duration.clean.Ce$disease.duration.years[duration.clean.Ce$sex=="M"]
y2 = duration.clean.Ce$HbB.Ce[duration.clean.Ce$sex=="M"]
x3 = duration.clean.Ce$disease.duration.years[duration.clean.Ce$sex=="F"]
y3 = duration.clean.Ce$HbB.Ce[duration.clean.Ce$sex=="F"]

plot (y~x, col="blue", main = "Cerebellum HbA", xlab="Duration (years)", ylab="HbA", ylim = c(0,(max(y,y1,y2,y3)+0.1)),
pch=20)
fit <- lm(y~x)
abline(fit, col="blue", lwd=2)
newx <- seq(min(x,x1,x2,x3), max(x,x1,x2,x3), 0.5)
prd <-predict(fit,newdata=data.frame(x=newx),interval = c("confidence"))
lines(newx,prd[,2],col="blue",lty=2)
lines(newx,prd[,3],col="blue",lty=2)
points (y1~x1, col="red",pch=20)
fit1 <- lm(y1~x1)
abline(fit1, col="red", lwd=2)
newx1 <- seq(min(x,x1,x2,x3), max(x,x1,x2,x3), 0.5)
prd1 <-predict(fit1,newdata=data.frame(x1=newx1),interval = c("confidence"))
lines(newx1,prd1[,2],col="red",lty=2)
lines(newx1,prd1[,3],col="red",lty=2)

plot (y2~x2, col="blue", main = "Cerebellum HbB", xlab="Duration (years)", ylab="HbB", ylim = c(0,(max(y,y1,y2,y3)+0.1)),
pch=20)
fit2 <- lm(y2~x2)
abline(fit, col="blue", lwd=2)
newx2 <- seq(min(x,x1,x2,x3), max(x,x1,x2,x3), 0.5)
prd2 <-predict(fit2,newdata=data.frame(x2=newx2),interval = c("confidence"))
lines(newx2,prd2[,2],col="blue",lty=2)
lines(newx2,prd2[,3],col="blue",lty=2)
points (y3~x3, col="red",pch=20)
fit3 <- lm(y3~x3)
abline(fit3, col="red", lwd=2)
newx3 <- seq(min(x,x1,x2,x3), max(x,x1,x2,x3), 0.5)
prd3 <-predict(fit3,newdata=data.frame(x3=newx3),interval = c("confidence"))
lines(newx3,prd3[,2],col="red",lty=2)
lines(newx3,prd3[,3],col="red",lty=2)
dev.off()



pdf("FL.sex.disease.duration.scatter.CI.bothsex.pdf")
par(mfrow = c(1,2))
x = duration.clean.FL$disease.duration.years[duration.clean.FL$sex=="M"]
y = duration.clean.FL$HbA.FL[duration.clean.FL$sex=="M"]
x1 = duration.clean.FL$disease.duration.years[duration.clean.FL$sex=="F"]
y1 = duration.clean.FL$HbA.FL[duration.clean.FL$sex=="F"]
x2 = duration.clean.FL$disease.duration.years[duration.clean.FL$sex=="M"]
y2 = duration.clean.FL$HbB.FL[duration.clean.FL$sex=="M"]
x3 = duration.clean.FL$disease.duration.years[duration.clean.FL$sex=="F"]
y3 = duration.clean.FL$HbB.FL[duration.clean.FL$sex=="F"]
```

```
[illegible]

plot (y~x, col="blue", main = "Frontal Lobe HbA", xlab="Duration (years)", ylab="HbA", ylim = c(0,
(max(y,y1,y2,y3)+0.1)),pch=20)
fit <- lm(y~x)
abline(fit, col="blue", lwd=2)
newx <- seq(min(x,x1,x2,x3), max(x,x1,x2,x3), 0.5)
prd <-predict(fit,newdata=data.frame(x=newx),interval = c("confidence"))
lines(newx,prd[,2],col="blue",lty=2)
lines(newx,prd[,3],col="blue",lty=2)
points (y1~x1, col="red",pch=20)
fit1 <- lm(y1~x1)
abline(fit1, col="red", lwd=2)
newx1 <- seq(min(x,x1,x2,x3), max(x,x1,x2,x3), 0.5)
prd1 <-predict(fit1,newdata=data.frame(x1=newx1),interval = c("confidence"))
lines(newx1,prd1[,2],col="red",lty=2)
lines(newx1,prd1[,3],col="red",lty=2)

plot (y2~x2, col="blue", main = "Frontal Lobe HbB", xlab="Duration (years)", ylab="HbB", ylim = c(0,
(max(y,y1,y2,y3)+0.1)),pch=20)
fit2 <- lm(y2~x2)
abline(fit, col="blue", lwd=2)
newx2 <- seq(min(x,x1,x2,x3), max(x,x1,x2,x3), 0.5)
prd2 <-predict(fit2,newdata=data.frame(x2=newx2),interval = c("confidence"))
lines(newx2,prd2[,2],col="blue",lty=2)
lines(newx2,prd2[,3],col="blue",lty=2)
points (y3~x3, col="red",pch=20)
fit3 <- lm(y3~x3)
abline(fit3, col="red", lwd=2)
newx3 <- seq(min(x,x1,x2,x3), max(x,x1,x2,x3), 0.5)
prd3 <-predict(fit3,newdata=data.frame(x3=newx3),interval = c("confidence"))
lines(newx3,prd3[,2],col="red",lty=2)
lines(newx3,prd3[,3],col="red",lty=2)
dev.off()



pdf("SN.sex.disease.duration.scatter.CI.bothsex.pdf")
par(mfrow = c(1,2))
x = duration.clean.SN$disease.duration.years[duration.clean.SN$sex=="M"]
y = duration.clean.SN$HbA.SN[duration.clean.SN$sex=="M"]
x1 = duration.clean.SN$disease.duration.years[duration.clean.SN$sex=="F"]
y1 = duration.clean.SN$HbA.SN[duration.clean.SN$sex=="F"]
x2 = duration.clean.SN$disease.duration.years[duration.clean.SN$sex=="M"]
y2 = duration.clean.SN$HbB.SN[duration.clean.SN$sex=="M"]
x3 = duration.clean.SN$disease.duration.years[duration.clean.SN$sex=="F"]
y3 = duration.clean.SN$HbB.SN[duration.clean.SN$sex=="F"]

plot (y~x, col="blue", main = "Substantia Nigra HbA", xlab="Duration (years)", ylab="HbA", ylim = c(0,
(max(y,y1,y2,y3)+0.1)),pch=20)
fit <- lm(y~x)
abline(fit, col="blue", lwd=2)
newx <- seq(min(x,x1,x2,x3), max(x,x1,x2,x3), 0.5)
prd <-predict(fit,newdata=data.frame(x=newx),interval = c("confidence"))
lines(newx,prd[,2],col="blue",lty=2)
lines(newx,prd[,3],col="blue",lty=2)
points (y1~x1, col="red",pch=20)
fit1 <- lm(y1~x1)
abline(fit1, col="red", lwd=2)
newx1 <- seq(min(x,x1,x2,x3), max(x,x1,x2,x3), 0.5)
prd1 <-predict(fit1,newdata=data.frame(x1=newx1),interval = c("confidence"))
lines(newx1,prd1[,2],col="red",lty=2)
lines(newx1,prd1[,3],col="red",lty=2)

plot (y2~x2, col="blue", main = "Substantia Nigra HbB", xlab="Duration (years)", ylab="HbB", ylim = c(0,
(max(y,y1,y2,y3)+0.1)),pch=20)
fit2 <- lm(y2~x2)
abline(fit, col="blue", lwd=2)
newx2 <- seq(min(x,x1,x2,x3), max(x,x1,x2,x3), 0.5)
prd2 <-predict(fit2,newdata=data.frame(x2=newx2),interval = c("confidence"))
lines(newx2,prd2[,2],col="blue",lty=2)
lines(newx2,prd2[,3],col="blue",lty=2)
points (y3~x3, col="red",pch=20)
fit3 <- lm(y3~x3)
abline(fit3, col="red", lwd=2)
newx3 <- seq(min(x,x1,x2,x3), max(x,x1,x2,x3), 0.5)
prd3 <-predict(fit3,newdata=data.frame(x3=newx3),interval = c("confidence"))
lines(newx3,prd3[,2],col="red",lty=2)
lines(newx3,prd3[,3],col="red",lty=2)
dev.off()
#######################################################################
########################################################################
library("ggplot2")
data<-read.csv(file = "normalised ratios to actin.csv", header = T)
data<-data[1:71,]

#create 4 PMI categories
hist(data[,5])
attach(data)
data$PMIcat[PMI > 30] <- "4"
data$PMIcat[PMI > 20 & PMI <= 30] <- "3"
data$PMIcat[PMI > 10 & PMI <= 20] <- "2"
data$PMIcat[PMI <= 10] <- "1"
```

```
data$PMIcat[PMI < 10] <- 1
detach(data)

#create 2 diagnosis categories
attach(data)
data$PD[diagnosis=="control"] <- "control"
data$PD[diagnosis=="PD1"] <- "PD"
data$PD[diagnosis=="PD2"] <- "PD"
data$PD[diagnosis=="PD3"] <- "PD"
detach(data)

#scatterplots Hb vs disease duration, coloured by gender, shaped by PMI
tiff("Cer HbB.tiff")
ggplot(data=data,aes(x=disease.duration..years., y=Cer.HbB, colour = Gender, shape = PMIcat)) +
  geom_point(alpha=0.4, size=4) +
  xlab("Disease duration (yr)") + ylab("mito/cyto HbB") + ggtitle("Cer HbB")+
  scale_colour_manual(values =c("#CC0000", "#0000CC"))+
  theme_classic() +
  theme(legend.position="right")+
  theme(plot.title=element_text(face="bold", size=20),
        axis.title.x = element_text(face="bold", size=15),
        axis.title.y = element_text(face="bold", size=15))
dev.off()

#PMI vs Cer HbB
tiff("PMI vs Cer HbB.tiff")
ggplot(data=data,aes(x=PMI, y=Cer.HbB,colour=Gender)) +
  geom_point(alpha=0.4, size=4) +
  geom_smooth(method = lm)+
  xlab("PMI(hr)") + ylab("HbB") + ggtitle("Cer HbB")+
  scale_colour_manual(values =c("#CC0000", "#0000CC"))+
  theme_classic() +
  theme(plot.title=element_text(face="bold", size=20),
        axis.title.x = element_text(face="bold", size=15),
        axis.title.y = element_text(face="bold", size=15))
theme(legend.position="right")
dev.off()

#PMI vs mitochondrial marker plots
tiff("PMI vs Ctx COXIV.tiff")
ggplot(data=data,aes(x=PMI, y=Ctx.COXIV,colour=Gender)) +
  geom_point(alpha=0.4, size=4) +
  geom_smooth(method = lm)+
  xlab("PMI(hr)") + ylab("COXIV") + ggtitle("Ctx COXIV")+
  scale_colour_manual(values =c("#CC0000", "#0000CC"))+
  theme_classic() +
  theme(plot.title=element_text(face="bold", size=20),
        axis.title.x = element_text(face="bold", size=15),
        axis.title.y = element_text(face="bold", size=15))
theme(legend.position="right")
dev.off()

tiff("PMI vs Cer COXIV.tiff")
ggplot(data=data,aes(x=PMI, y=Cer.COXIV,colour=Gender)) +
  geom_point(alpha=0.4, size=4) +
  geom_smooth(method = lm)+
  xlab("PMI(hr)") + ylab("COXIV") + ggtitle("Cer COXIV")+
  scale_colour_manual(values =c("#CC0000", "#0000CC"))+
  theme_classic() +
  theme(plot.title=element_text(face="bold", size=20),
        axis.title.x = element_text(face="bold", size=15),
        axis.title.y = element_text(face="bold", size=15))
theme(legend.position="right")
dev.off()

tiff("PMI vs SN COXIV.tiff")
ggplot(data=data,aes(x=PMI, y=SN.COXIV,colour=Gender)) +
  geom_point(alpha=0.4, size=4) +
  geom_smooth(method = lm)+
  xlab("PMI(hr)") + ylab("COXIV") + ggtitle("SN COXIV")+
  scale_colour_manual(values =c("#CC0000", "#0000CC"))+
  theme_classic() +
  theme(plot.title=element_text(face="bold", size=20),
        axis.title.x = element_text(face="bold", size=15),
        axis.title.y = element_text(face="bold", size=15))
theme(legend.position="right")
dev.off()

#a-syn pathology vs diagnosis
tiff("a.syn.tiff")
ggplot(data, aes(x=diagnosis, y=a.syn))+
  geom_boxplot()+
  xlab("")+
  ylab("alpha synuclein")+
  ggtitle("")+
  guides(fill=FALSE)+
  scale_fill_manual(values=c("grey","white"))+
  theme_classic()+
  theme(plot.title=element_text(face="bold", size=20),
        axis.text.x = element_text(face="bold", size=15),
        axis.title.y = element_text(face="bold", size=15))
dev.off()
```

```
#matched sample data
data2<-read.csv(file = "PD data matched samples.csv", header = T)
tiff("Ctx HbA matched samples.tiff")
ggplot(data=data2,aes(x=disease.duration..years., y=Ctx.HbA,colour=Gender)) +
  geom_point(alpha=0.4, size=4) +
  geom_smooth(method = lm)+
  xlab("Disease duration (yr)") + ylab("mito/cyto HbA") + ggtitle("Ctx HbA")+
  scale_colour_manual(values =c("#CC0000", "#0000CC"))+
  theme_classic() +
  theme(plot.title=element_text(face="bold", size=20),
        axis.title.x = element_text(face="bold", size=15),
        axis.title.y = element_text(face="bold", size=15))+
  theme(legend.position="right")
dev.off()
tiff("Ctx HbB matched samples.tiff")
ggplot(data=data2,aes(x=disease.duration..years., y=Ctx.HbB,colour=Gender)) +
  geom_point(alpha=0.4, size=4) +
  geom_smooth(method = lm)+
  xlab("Disease duration (yr)") + ylab("mito/cyto HbB") + ggtitle("Ctx HbB")+
  scale_colour_manual(values =c("#CC0000", "#0000CC"))+
  theme_classic() +
  theme(plot.title=element_text(face="bold", size=20),
        axis.title.x = element_text(face="bold", size=15),
        axis.title.y = element_text(face="bold", size=15))+
  theme(legend.position="right")
dev.off()
tiff("Cer HbA matched samples.tiff")
ggplot(data=data2,aes(x=disease.duration..years., y=Cer.HbA,colour=Gender)) +
  geom_point(alpha=0.4, size=4) +
  geom_smooth(method = lm)+
  xlab("Disease duration (yr)") + ylab("mito/cyto HbA") + ggtitle("Cer HbA")+
  scale_colour_manual(values =c("#CC0000", "#0000CC"))+
  theme_classic() +
  theme(plot.title=element_text(face="bold", size=20),
        axis.title.x = element_text(face="bold", size=15),
        axis.title.y = element_text(face="bold", size=15))+
  theme(legend.position="right")
dev.off()
tiff("Cer HbB matched samples.tiff")
ggplot(data=data2,aes(x=disease.duration..years., y=Cer.HbB,colour=Gender)) +
  geom_point(alpha=0.4, size=4) +
  geom_smooth(method = lm)+
  xlab("Disease duration (yr)") + ylab("mito/cyto HbB") + ggtitle("Cer HbB")+
  scale_colour_manual(values =c("#CC0000", "#0000CC"))+
  theme_classic() +
  theme(plot.title=element_text(face="bold", size=20),
        axis.title.x = element_text(face="bold", size=15),
        axis.title.y = element_text(face="bold", size=15))+
  theme(legend.position="right")
dev.off()
tiff("SN HbA matched samples.tiff")
ggplot(data=data2,aes(x=disease.duration..years., y=SN.HbA,colour=Gender)) +
  geom_point(alpha=0.4, size=4) +
  geom_smooth(method = lm)+
  xlab("Disease duration (yr)") + ylab("mito/cyto HbA") + ggtitle("SN HbA")+
  scale_colour_manual(values =c("#CC0000", "#0000CC"))+
  theme_classic() +
  theme(plot.title=element_text(face="bold", size=20),
        axis.title.x = element_text(face="bold", size=15),
        axis.title.y = element_text(face="bold", size=15))+
  theme(legend.position="right")
dev.off()
tiff("SN HbB matched samples.tiff")
ggplot(data=data2,aes(x=disease.duration..years., y=SN.HbB,colour=Gender)) +
  geom_point(alpha=0.4, size=4) +
  geom_smooth(method = lm)+
  xlab("Disease duration (yr)") + ylab("mito/cyto HbB") + ggtitle("SN HbB")+
  scale_colour_manual(values =c("#CC0000", "#0000CC"))+
  theme_classic() +
  theme(plot.title=element_text(face="bold", size=20),
        axis.title.x = element_text(face="bold", size=15),
        axis.title.y = element_text(face="bold", size=15))+
  theme(legend.position="right")
dev.off()
#plot 5 random M vs random F samples Cer HbB vs disease duration
male <-subset(data, Gender=="M" & PD=="PD",select=sample.ID:PD)
female <-subset(data, Gender=="F"& PD=="PD",select=sample.ID:PD)
randommale <- male[sample(1:nrow(male), 5,
                          replace=FALSE),]
randomfemale <- female[sample(1:nrow(female), 5,
                              replace=FALSE),]
random <-rbind(randommale,randomfemale)
tiff("random 5Mvs5F CerHbB vs disease duration.tiff")
ggplot(data=random,aes(x=disease.duration..years., y=Cer.HbB,colour=Gender)) +
  geom_point(alpha=0.4, size=1.75) +
  geom_smooth(method = lm)+
  xlab("duration (years)") + ylab("HbB") + ggtitle("Cer HbB - random10")+
  theme_bw() +
  theme(legend.position="right")
dev.off()
```
