# Supplementary material for: Analysis of Mitochondrial haemoglobin in Parkinson's disease brain
Source: Mitochondrion. 2016 Jul;29:45–52. doi: 10.1016/j.mito.2016.05.001 (PMC4940210; doi:10.1016/j.mito.2016.05.001)
Supplement: Supplementary Table 2 — Full western blot densitometry dataset. [file mmc5.docx]

| sample ID | diagnosis | age | Gender | PMI | a-syn | disease duration (years) | Ctx HbA | Ctx HbB | Ctx COXIV | Cer HbA | Cer HbB | Cer COXIV | SN HbA | SN HbB | SN COXIV |
| --- | --- | --- | --- | --- | --- | --- | --- | --- | --- | --- | --- | --- | --- | --- | --- |
| CO25 | control | 35 | M | 22 | NA | NA | 0.35 | 0.45 | 3.8 | 0.5 | 0.68 | 8.5 | NA | NA | NA |
| 62/02 | control | 58 | M | NA | NA | NA | 0.1 | 0.29 | 1.64 | 0.45 | 0.44 | 4.93 | NA | NA | NA |
| PDC005 | control | 58 | M | 9 | NA | NA | 0.75 | NA | 16.15 | 0.87 | 1.36 | 10.71 | 1.11 | NA | 20.9 |
| 75/02 | control | 61 | F | NA | NA | NA | 0.34 | 0.4 | 2.36 | 0.28 | 0.39 | 4.45 | NA | NA | NA |
| 85/07 | control | 63 | M | NA | NA | NA | 0.18 | 0.29 | 3.13 | NA | NA | NA | NA | NA | NA |
| PDC022 | control | 65 | M | 12 | NA | NA | 0.97 | 0.61 | 21.25 | 0.69 | 0.59 | 22.7 | 0.42 | 0.82 | 0.8 |
| CO36 | control | 68 | M | 30 | NA | NA | 0.82 | 0.89 | 8.3 | 0.81 | 0.7 | 18.8 | 0.46 | 0.49 | 13.5 |
| CO48 | control | 68 | M | 10 | NA | NA | 0.65 | 0.75 | 15.5 | 0.51 | 0.65 | 10.9 | 0.47 | 0.59 | 24.6 |
| CO22 | control | 69 | F | 33 | NA | NA | 0.63 | 0.78 | 7.3 | 0.65 | 0.56 | 2.4 | 0.6 | 0.39 | 6.2 |
| PDC008 | control | 71 | F | 17 | NA | NA | 1.11 | 1 | 45.6 | 0.75 | 1.09 | 26.2 | 0.82 | 0.17 | 17.4 |
| 10/04' | control | 73 | M | NA | NA | NA | 0.16 | 0.36 | 5.56 | NA | NA | NA | NA | NA | NA |
| 50/06 | control | 75 | M | NA | NA | NA | 0.18 | 0.25 | 1.42 | NA | NA | NA | NA | NA | NA |
| 17/04' | control | 75 | F | NA | NA | NA | 0.19 | 0.39 | 15.17 | NA | NA | NA | NA | NA | NA |
| 106/03 | control | 77 | F | NA | NA | NA | 0.33 | 0.39 | 3.27 | NA | NA | NA | NA | NA | NA |
| PDC030 | control | 77 | M | 17 | NA | NA | 1.06 | 1.1 | 8.1 | 0.54 | 0.61 | 4.8 | 0.9 | 0.72 | 7.9 |
| CO45 | control | 77 | M | 22 | NA | NA | 0.37 | 0.4 | 25.5 | 0.53 | 0.65 | 2.1 | 0.64 | 0.55 | 7.6 |
| 36/04 | control | 78 | M | NA | NA | NA | 0.29 | 0.46 | 14.49 | NA | NA | NA | NA | NA | NA |
| PDC023 | control | 78 | F | 23 | NA | NA | 0.58 | 0.76 | NA | 0.42 | 0.44 | NA | NA | NA | NA |
| CO26 | control | 78 | F | 33 | NA | NA | 0.22 | 0.15 | 4.3 | 0.68 | 0.28 | 7.5 | 0.95 | 0.51 | 2.8 |
| 98/07 | control | 80 | M | NA | NA | NA | 0.56 | 0.47 | 4.15 | 0.18 | 0.3 | 7.95 | NA | NA | NA |
| PDC026 | control | 80 | F | 23 | NA | NA | 1.36 | 0.98 | 14.4 | 0.35 | 0.33 | 24.4 | NA | NA | NA |
| 61/07 | control | 81 | M | NA | NA | NA | 0.38 | 0.32 | 4.4 | 0.28 | 0.81 | 7.11 | NA | NA | NA |
| 21/04' | control | 81 | M | NA | NA | NA | 0.73 | 0.6 | 7.01 | NA | NA | NA | NA | NA | NA |
| PDC029 | control | 82 | M | 48 | NA | NA | 0.7 | 0.59 | 7.5 | 0.65 | 0.7 | 12.3 | 0.74 | 0.74 | 6.3 |
| CO15 | control | 82 | M | 21 | NA | NA | 1.07 | 0.9 | 3.1 | 0.71 | 0.62 | 5.8 | 0.33 | 0.56 | 0.12 |
| CO39 | control | 82 | M | 21 | NA | NA | 0.86 | 1.23 | 11.6 | 1.27 | 0.38 | 30.6 | NA | NA | NA |
| PDC028 | control | 84 | F | 11 | NA | NA | 0.75 | 0.92 | 7.65 | 0.58 | 1.49 | 0.55 | 0.73 | 0.95 | 1.25 |
| CO37 | control | 84 | M | 5 | NA | NA | 0.81 | 1.3 | 3.5 | 1 | 0.97 | 6.7 | NA | NA | 5.4 |
| CO32 | control | 88 | M | 22 | NA | NA | 1.07 | 1.19 | 21.9 | 0.98 | 0.77 | 15.8 | 0.65 | 0.57 | 38.6 |
| PDC034 | control | 90 | M | 12 | NA | NA | 0.93 | 0.97 | 15.8 | 0.58 | 0.68 | 5.3 | 0.78 | 0.92 | 1.3 |
| PDC016 | control | 93 | F | 22 | NA | NA | 0.52 | 0.88 | 26.8 | 0.85 | 0.76 | 10.5 | 0.76 | 0.71 | 15.98 |
| 002/08 | PD1 | 69 | M | NA | NA | 1 | 0.39 | 0.44 | 11.04 | 0.75 | 0.6 | 11.95 | NA | NA | NA |
| 196/01 | PD1 | 71 | M | NA | NA | 1 | 0.83 | 0.71 | 8.83 | NA | NA | NA | NA | NA | NA |
| PD131 | PD1 | 76 | F | 22 | 6 | 11 | 1.27 | 1.13 | 62 | 0.7 | 1.51 | 34.5 | 1.37 | 0.12 | 7.33 |
| 044/07 | PD1 | 78 | M | NA | NA | 6 | 0.43 | 0.47 | 4.58 | 0.3 | 0.37 | 4.21 | NA | NA | NA |
| PD079 | PD1 | 78 | F | 22 | 6 | 19 | 0.84 | 1.7 | NA | 0.91 | 0.77 | NA | NA | NA | NA |
| PD014 | PD1 | 79 | M | 21 | 3 | 12 | 1.07 | 1.48 | 18.4 | 0.26 | 0.48 | 12.6 | 0.74 | 0.55 | 5.8 |
| PD045 | PD1 | 80 | M | 16 | 6 | 19 | 0.91 | 0.75 | 12.3 | 0.57 | 0.58 | 6.14 | 0.63 | 0.41 | 0.6 |
| PD063 | PD1 | 80 | F | 10 | 4 | 13 | 1.01 | 0.69 | 31.07 | 1.03 | 0.71 | 19.69 | 0.74 | 0.76 | 4.53 |
| PD028 | PD1 | 82 | M | 14 | 6 | 18 | 1.18 | 1.27 | 13.3 | 0.67 | 0.78 | 8.4 | 0.82 | 0.58 | 1.84 |
| PD050 | PD1 | 82 | F | 18 | 6 | 14 | 0.77 | 0.52 | 7.36 | 0.7 | 0.68 | 8.75 | 1.16 | 0.8 | 27.97 |
| PD099 | PD1 | 82 | M | 10 | 6 | 11 | 0.6 | 0.49 | NA | 0.83 | 0.53 | NA | 1.19 | 0.45 | NA |
| PD124 | PD1 | 82 | F | 13 | 6 | 17 | 0.85 | 0.98 | 40.5 | 1.38 | 1.31 | 7.6 | 0.76 | 2.39 | 43.1 |
| PD016 | PD1 | 85 | F | 14 | 6 | 18 | 1 | 0.81 | 26.4 | 0.52 | 0.57 | 5.7 | 0.34 | 0.11 | 0.7 |
| PD121 | PD2 | 69 | M | 9 | 6 | 4 | 0.82 | 0.7 | 15.26 | 0.88 | 0.8 | 3.33 | 0.87 | 0.72 | 3.18 |
| PD109 | PD2 | 72 | M | 9 | 4 | 6 | 0.72 | 1.07 | 10.13 | 0.97 | 0.81 | 5.22 | 0.67 | 0.56 | 5.51 |
| 118/03 | PD2 | 73 | M | NA | NA | 1 | 0.22 | 0.22 | 5.46 | 0.23 | 0.39 | 3.86 | NA | NA | NA |
| PD081 | PD2 | 73 | M | 19 | 6 | 9 | 0.73 | 0.51 | 22.8 | 0.83 | 0.65 | 15.3 | 0.74 | 0.53 | 3.4 |
| PD036 | PD2 | 76 | M | 10 | 3 | 10 | 0.83 | 0.89 | NA | 0.72 | 1 | 12.5 | 0.53 | 0.65 | NA |
| PD041 | PD2 | 77 | M | 6 | 6 | 10 | 0.29 | 0.31 | 33.1 | 0.68 | 0.78 | 4.6 | 0.8 | 0.33 | NA |
| PD007 | PD2 | 78 | M | 22 | 3 | 10 | 1.09 | 1.22 | 40.45 | 0.82 | 0.74 | 3.4 | 1.07 | 0.59 | 4.7 |
| 025/08 | PD2 | 79 | F | NA | NA | 6 | 0.5 | 0.49 | 4.46 | NA | NA | NA | NA | NA | NA |
| 64/03 | PD2 | 80 | M | NA | NA | 10 | 0.3 | 0.57 | 5.25 | NA | NA | NA | NA | NA | NA |
| PD051 | PD2 | 80 | M | 7 | 5 | 5 | 1.17 | 1.01 | 12.41 | 0.91 | 0.95 | 8.33 | NA | NA | NA |
| 05/07' | PD2 | 81 | F | NA | NA | 1 | 0.6 | 0.4 | 10.69 | NA | NA | NA | NA | NA | NA |
| PD023 | PD2 | 82 | M | 28 | 6 | 7 | 0.94 | 0.67 | 13.67 | 0.96 | 0.56 | 3.49 | 0.64 | 0.13 | 7.28 |
| PD067 | PD2 | 83 | M | 10 | 6 | 9 | 0.62 | 0.81 | 17.7 | 0.51 | 0.63 | 24.04 | 0.8 | 0.54 | 6.33 |
| PD086 | PD2 | 87 | F | 22 | 4 | 9 | 0.6 | 0.77 | 24.96 | 0.88 | 0.86 | 20.1 | 0.64 | 0.51 | 9.04 |
| PD077 | PD3 | 58 | M | 18 | 6 | 15 | 0.77 | 0.53 | 8.31 | 0.53 | 0.46 | 2.55 | 0.68 | 0.55 | 0.63 |
| 13/07' | PD3 | 59 | M | NA | NA | 12 | 0.56 | 0.46 | 4.04 | NA | NA | NA | NA | NA | NA |
| PD115 | PD3 | 63 | M | 21 | 6 | 9 | 0.78 | 1.01 | NA | 0.73 | 0.54 | NA | 0.88 | 0.66 | 5.39 |
| 028/05 | PD3 | 64 | M | NA | NA | 3 | 0.23 | 0.38 | 4.59 | 0.27 | 0.6 | 3.96 | NA | NA | NA |
| PD258 | PD3 | 69 | M | 10 | 6 | 17 | 0.93 | 1.17 | 11.78 | 0.78 | 0.9 | 3.74 | 0.88 | 0.72 | 4.95 |
| 104/07 | PD3 | 70 | M | NA | NA | 11 | 0.3 | 0.3 | 11.04 | 0.34 | 0.39 | 11.95 | NA | NA | NA |
| PD268 | PD3 | 72 | M | 8 | 6 | 20 | 0.81 | 0.67 | 52.1 | 0.75 | 0.53 | 28.5 | 0.64 | 0.22 | 6.45 |
| PD125 | PD3 | 74 | M | 20 | 6 | 25 | 0.53 | 1.29 | NA | 0.77 | 0.99 | 21.89 | 0.72 | 0.65 | 14.92 |
| PD142 | PD3 | 74 | M | 3 | 5 | 21 | 0.79 | 0.38 | 17.19 | 0.46 | 0.75 | 25.62 | 0.46 | 0.54 | 19.02 |
| PD020 | PD3 | 75 | M | 2 | 6 | 34 | 0.98 | 0.98 | 29.99 | 0.67 | 0.58 | 6.21 | 1.23 | 1.5 | 5.57 |
| PD104 | PD3 | 75 | M | 15 | 6 | 25 | 0.85 | 0.76 | 2.95 | 0.84 | 0.6 | 2.6 | 0.67 | 0.46 | 1.6 |
| PD021 | PD3 | 76 | M | 17 | 6 | 27 | 0.82 | 0.81 | 25.77 | 0.51 | 0.45 | 4.53 | NA | NA | NA |
| PD117 | PD3 | 77 | F | 6 | 5 | 31 | 0.46 | 1.08 | 13.24 | 0.68 | 0.32 | 7.01 | 0.89 | 0.48 | 3.72 |
