# Supplementary material for: Analysis of Mitochondrial haemoglobin in Parkinson's disease brain
Source: Mitochondrion. 2016 Jul;29:45–52. doi: 10.1016/j.mito.2016.05.001 (PMC4940210; doi:10.1016/j.mito.2016.05.001)
Supplement: Supplementary Table 3 — Accumulated analysis of variance. Sequential regression analysis was performed using Genstat, using the general linear regression tool, and adding the variables to the model in order with the largest mean square first. [file mmc6.docx]

| Change | d.f. | s.s. | m.s. | v.r. | F pr. |
| --- | --- | --- | --- | --- | --- |
| Disease duration | 1 | 0.14279 | 0.14279 | 2.47 | 0.128 |
| Gender | 1 | 0.17546 | 0.17546 | 3.03 | 0.093 |
| PMI | 1 | 0.00858 | 0.00858 | 0.15 | 0.703 |
| Residual | 26 | 1.50456 | 0.05787 |  |  |
| Total | 29 | 1.83139 | 0.06315 |  |  |

Supplementary Table 3. Accumulated analysis of variance. Sequential regression analysis was performed using Genstat, using the general linear regression tool, and adding the variables to the model in order with the largest mean square first.
