# Supplementary material for: Analysis of Mitochondrial haemoglobin in Parkinson's disease brain
Source: Mitochondrion. 2016 Jul;29:45–52. doi: 10.1016/j.mito.2016.05.001 (PMC4940210; doi:10.1016/j.mito.2016.05.001)
Supplement: Supplementary Table 4 — Slide scanner settings for immunofluorescence. [file mmc7.docx]

|  |  | Installed in conventional cube within the slide scanner | | |
| --- | --- | --- | --- | --- |
|  | Single Band pass excitation filter (installed in the Lumencor SpectraX) | Multi-Band pass exciter | Multi Band pass Dichroic | Multi Band pass Emission filter |
| DAPI | [FF01-386/23-25](http://www.semrock.com/FilterDetails.aspx?id=FF01-386/23-25) | Top of Form  [FF01-387/485/559/649-25](http://www.semrock.com/FilterDetails.aspx?id=FF01-387/485/559/649-25) | Top of Form  [FF410/504/582/669-Di01-25x36](http://www.semrock.com/FilterDetails.aspx?id=FF410/504/582/669-Di01-25x36) | Top of Form  [FF01-440/521/607/700-25](http://www.semrock.com/FilterDetails.aspx?id=FF01-440/521/607/700-25) |
| Alexa 488 | [FF02-485/20-25](http://www.semrock.com/FilterDetails.aspx?id=FF02-485/20-25) | Shared with above | Shared with above | Shared with above |
| Alexa 532 | [FF01-550/88-25](http://www.semrock.com/FilterDetails.aspx?id=FF01-550/88-25) | Shared with above | Shared with above | Shared with above |
