# Supplementary material for: Analysis of Mitochondrial haemoglobin in Parkinson's disease brain
Source: Mitochondrion. 2016 Jul;29:45–52. doi: 10.1016/j.mito.2016.05.001 (PMC4940210; doi:10.1016/j.mito.2016.05.001)
Supplement: Supplementary Table 5 — Matched samples ‘equal numbers of male and female brains’, used to generate Supplementary Fig. 6. [file mmc8.docx]

| **ID** | **Diagnosis** | **Age (yr)** | **Sex** | **Disease duration (yr)** | **α-syn** | **Cause of death** | **Post mortem delay (hr)** | **Ctx** | **Cer** | **SN** |
| --- | --- | --- | --- | --- | --- | --- | --- | --- | --- | --- |
| PD131 | Late PD | 76 | F | 11 | 6 | Not reported | 22 |  |  |  |
| PD014 | Late PD | 79 | M | 12 | 3 | Parkinson’s | 21 |  |  |  |
| PD045 | Late PD | 80 | M | 19 | 6 | Not reported | 16 |  |  |  |
| PD063 | Late PD | 80 | F | 13 | 4 | Old age and Parkinson’s | 10 |  |  |  |
| PD028 | Late PD | 82 | M | 18 | 6 | Not reported | 14 |  |  |  |
| PD050 | Late PD | 82 | F | 14 | 6 | Chest infection & CVA | 18 |  |  |  |
| PD099 | Late PD | 82 | M | 11 | 6 | Pneumonia | 10 |  |  |  |
| PD124 | Late PD | 82 | F | 17 | 6 | Unknown | 13 |  |  |  |
| PD016 | Late PD | 85 | F | 18 | 6 | Bronchopneumonia/PD | 14 |  |  |  |
| PD023 | Early PD | 82 | M | 7 | 6 | Unknown | 28 |  |  |  |
| PD067 | Early PD | 83 | M | 9 | 6 | Not reported | 10 |  |  |  |
| PD086 | Early PD | 87 | F | 9 | 4 | GI bleeding | 22 |  |  |  |
| PD020 | Young PD | 75 | M | 34 | 6 | Not reported | 2 |  |  |  |
| PD117 | Young PD | 77 | F | 31 | 5 | Unknown | 6 |  |  |  |
